# Entomopathogenic nematode-associated microbiota: from monoxenic paradigm to pathobiome

**DOI:** 10.1186/s40168-020-00800-5

**Published:** 2020-02-24

**Authors:** Jean-Claude Ogier, Sylvie Pagès, Marie Frayssinet, Sophie Gaudriault

**Affiliations:** IDGIMI, INRAe-Université de Montpellier, 34095 Montpellier, France

**Keywords:** Entomopathogenic nematode, Insect disease, Microbiota, Multigenic metabarcoding, *Xenorhabdus*, *Pseudomonas*, Pathobiome

## Abstract

**Background:**

The holistic view of bacterial symbiosis, incorporating both host and microbial environment, constitutes a major conceptual shift in studies deciphering host-microbe interactions. Interactions between *Steinernema* entomopathogenic nematodes and their bacterial symbionts, *Xenorhabdus*, have long been considered monoxenic two partner associations responsible for the killing of the insects and therefore widely used in insect pest biocontrol. We investigated this “monoxenic paradigm” by profiling the microbiota of infective juveniles (IJs), the soil-dwelling form responsible for transmitting *Steinernema*-*Xenorhabdus* between insect hosts in the parasitic lifecycle.

**Results:**

Multigenic metabarcoding (16S and *rpoB* markers) showed that the bacterial community associated with laboratory-reared IJs from *Steinernema carpocapsae*, *S*. *feltiae*, *S*. *glaseri* and *S*. *weiseri* species consisted of several Proteobacteria. The association with *Xenorhabdus* was never monoxenic. We showed that the laboratory-reared IJs of *S*. *carpocapsae* bore a bacterial community composed of the core symbiont (*Xenorhabdus nematophila*) together with a frequently associated microbiota (FAM) consisting of about a dozen of Proteobacteria (*Pseudomonas*, *Stenotrophomonas*, *Alcaligenes*, *Achromobacter*, *Pseudochrobactrum*, *Ochrobactrum*, *Brevundimonas*, *Deftia*, etc.). We validated this set of bacteria by metabarcoding analysis on freshly sampled IJs from natural conditions. We isolated diverse bacterial taxa, validating the profile of the *Steinernema* FAM. We explored the functions of the FAM members potentially involved in the parasitic lifecycle of *Steinernema*. Two species, *Pseudomonas protegens* and *P*. *chlororaphis*, displayed entomopathogenic properties suggestive of a role in *Steinernema* virulence and membership of the *Steinernema* pathobiome.

**Conclusions:**

Our study validates a shift from monoxenic paradigm to pathobiome view in the case of the *Steinernema* ecology. The microbial communities of low complexity associated with EPNs will permit future microbiota manipulation experiments to decipher overall microbiota functioning in the infectious process triggered by EPN in insects and, more generally, in EPN ecology.

## Background

Host-microbe symbioses, which range from mutualistic to pathogenic interactions, are ubiquitous in both marine and terrestrial environments. The host microbiota and its variations have recently been shown to be important for the outcome of interaction between symbiotic microbes and plants [1], vertebrates [2] or invertebrates [3]. When symbiotic microbes are transmitted by macro-organism hosts, symbiont dispersion may also be influenced by the cohort of micro-organisms carried by the host [4, 5]. If we are to understand host-microbe interactions, we must therefore consider the cohort of micro-organisms associated with the host.

The pathobiome is defined as the pathogenic agent together with its overall microbial environment, which contributes to pathogenesis [6]. Within the context of mammalian gut microbiota, this concept leads to the definition of several new categories on the commensal-to-pathogen spectrum: accessory pathogens, which act synergistically, under certain conditions to enhance the virulence of the main pathogenic bacteria and pathobionts, which are generally benign within the indigenous community and become pathogenic when homeostasis is disrupted [7].

Invertebrates rely on symbionts for many of their life history traits [8, 9]. Moreover, their microbial communities can be analysed with methodologies that cannot be used on vertebrates. Invertebrates are therefore the favoured models for explorations of the pathobiome concept. For instance, experimental inoculations or transverse transplantations of gut microbiota in honey and bumble bees have been shown to lead to changes in susceptibility to the protozoan *Lotmaria passim* [10] and the trypanosomatid parasite *Crithidia bombi* [11]. In the lepidopteran *Spodoptera littoralis*, the silencing of host immune pathways by RNAi revealed that *Serratia* and *Clostridium* species in the gut microbiota switched from asymptomatic symbionts to haemocoel pathogens during *Bacillus thuringiensis* infection [12].

Invertebrates may themselves be parasites. The infectious process results not only from their own virulence, but also from their cohort of associated micro-organisms. Many examples are provided by parasitic helminths and nematodes in particular [13]. For instance, the obligate endosymbiont *Wolbachia* plays a key role in the pathogenesis of the filarial nematodes responsible for lymphatic filariasis and river blindness [14]. Other emblematic nematode-bacterium associations include those between entomopathogenic nematodes *Heterorhabditis* and *Steinernema* and the ϒ-Proteobacteria *Photorhabdus* and *Xenorhabdus*, which inhabit their gut. The bacteria are involved in both insect killing and the lifecycle of the nematodes [15].

*Steinernema* and its intestinal symbiotic bacterium, *Xenorhabdus*, have long constituted a model system for investigating pathogenesis and mutualism [16–18]. They establish a sustainable association in lifecycles with three main steps [16–18]. The infective juveniles (IJs) of *Steinernema* are the free soil-dwelling forms of the nematode harbouring the symbiotic bacteria in their gut (step 1). On encountering a living insect larva, the IJ enters the insect gut and perforates the insect gut wall, which allows the injection of the *Xenorhabdus* symbiont into the insect hemocoel (body cavity) before the passage of the IJ through the insect midgut [19]. In the hemolymph (insect blood), *Xenorhabdus* multiplies and subsequently kills the insect (step 2). The penetration of IJs also permits insect gut microbiota translocation and proliferation into the insect hemocoel [20]. Within the insect cadaver (step 3), *Xenorhabdus* bacteria contribute to nematode maturation and reproduction, by degrading the insect tissues, feeding the nematode and outcompeting soil micro-organisms for nutrient acquisition in the insect cadaver. Once the nutrient resources are depleted, new IJs are produced, which specifically re-associate with their cognate *Xenorhabdus* symbiont and return to the soil (step 1).

The *Steinernema* IJs, which are the vector of *Xenorhabdus* between insect hosts, have a number of unusual features. They are non-feeding third-stage larvae enclosed within the second-stage cuticle, which closes the intestinal orifices (mouth and anus) and is lost only when the nematodes reach the insect gut [19, 21, 22]. The *Xenorhabdus* symbiont is carried in a dedicated part of the anterior intestine of the IJ, known as the receptacle [23–25]. Observations of these anatomical and histological aspects led to the idea of natural monoxeny in the *Xenorhabdus*-*Steinernema* symbiosis [26]. However, several culture-based studies have reported associations of bacteria other than the cognate *Xenorhabdus* symbiont with *Steinernema* IJs [26–32]. Unidentified bacteria have been observed in the intercellular space between the second- and third-stage cuticles [26].

Community profiling has progressed considerably over the last 10 years, making it possible to circumvent some of the limitations of culture-based methods (see for example [33–35]). These methods have been used to describe the microbiota of the free soil-dwelling nematodes *Acrobeloides maximus* and *Caenorhabditis elegans* [36–38]. Next-generation sequencing has recently been used to profile bacterial community dynamics in the cadavers of insect larvae infected with the entomopathogenic nematode *Heterorhabditis*, but the bacterial community associated with the IJs has not been extensively studied [39], and such approaches have never been used on *Steinernema*.

In this study, we investigated the hypothetical monoxeny of the *Xenorhabdus*-*Steinernema* symbiotic interaction. We first used a metabarcoding approach to describe the microbial community associated with several *Steinernema* species reared in the laboratory. We then focused on the well-characterised *S*. *carpocapsae* nematode, for which we have access to diverse strains and multiplication batches from different laboratories. We provide the first demonstration that, in this model, bacteria other than the symbiont *X*. *nematophila* are sustainably associated with *S*. *carpocapsae* IJs. We validated the pertinence of this frequently associated microbiota (FAM) by performing metabarcoding analysis on IJs freshly isolated from their natural habitat, the soil. We isolated a large range of bacterial strains from the FAM and explored some of their possible functions in the parasitic lifecycle of *Steinernema*. Two of the species identified, *Pseudomonas protegens* and *Pseudomonas chlororaphis*, kill insects as efficiently as the cognate *Xenorhabdus* symbiont, suggesting that they may belong to the IJ *Steinernema* pathobiome.

## Results

**Fig. 1:**
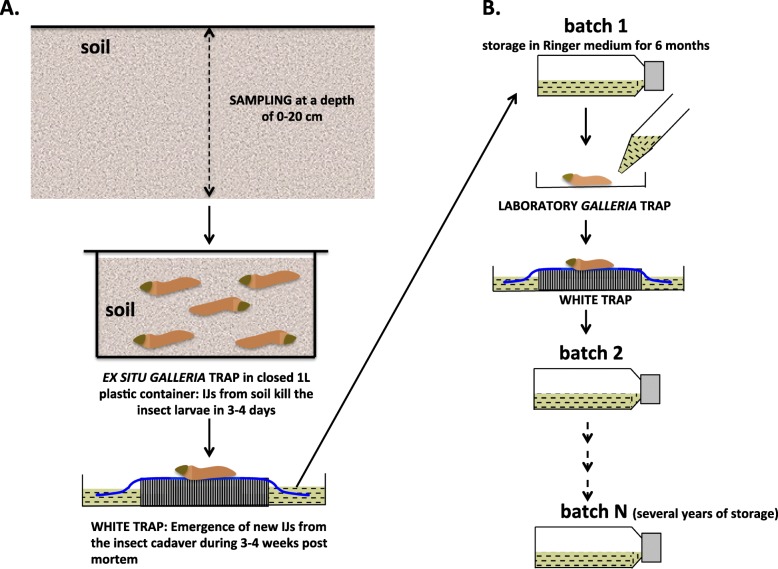
Ex situ isolation, storage and laboratory multiplication of *Steinernema* entomopathogenic nematodes. **a** Ex situ *Galleria* trap methodology. Soil was sampled at a depth of 0–20 cm and transferred to 1 L plastic containers. Five last-instar *Galleria mellonella* larvae were placed in each container (ex situ *Galleria* trap). The containers were stored in the dark at 18 °C. After 7 days, dead *G*. *mellonella* were placed in a White trap consisting of an overturned 35-mm Petri dish covered with a piece of material (in blue) and placed in a larger Petri dish containing Ringer solution. After the production of a few generations of nematodes in *G*. *mellonella* cadavers, the IJs left the cadaver and migrated into the Ringer solution via the wet material (black dashes in Ringer solution). **b** IJ storage and laboratory *Galleria* trap method. IJs emerging from *Steinernema* and *Heterorhabditis* species were stored in 250-mL flasks containing 80 mL of Ringer solution supplemented with 0.1% formaldehyde at 9 °C, and flasks containing 80 mL of Ringer solution supplemented with 0.01% formaldehyde at 15 °C, respectively. Every 6 months, stocks were multiplied by adding 50–100 IJs to *Galleria* larvae placed on filter paper in a Petri dish (laboratory *Galleria* trap). Once the insects had died, their cadavers were placed in a White trap, as described above. Multiplication batches were labelled with the infestation date

The IJs of *Steinernema* are living in soils but cannot directly be isolated from their habitat. They are first captured by ex situ *Galleria* trap (Fig. 1a). Once isolated, they are stored and multiplied on laboratory *Galleria* trap (Fig. 1b).

### Optimization of a metabarcoding pipeline for molecular description of the bacterial microbiota of *Steinernema* IJs reared in laboratory

DNA was extracted from an IJ nematode sample (*S*. *carpocapsae* SK27 batch 23_08_16; Additional file 1) from our laboratory stock, from controls (Kitome_QE, Kitome_MN, Tap water and Ringer) and from *Galleria* larvae. Amplification and sequencing of the V3V4 region of the 16S rRNA gene generated a total of 1,907,256 reads (mean of 45,411 reads per sample; number of samples = 42). Based on rarefaction curves, an adequate sequencing depth was reached at 15,000 reads per sample (Additional file 2). α-Diversity indicators for the bacterial communities associated with the IJs, *Galleria* and control samples highlighted the low diversity of the bacterial communities within the different samples (Additional file 3).

**Fig. 2:**
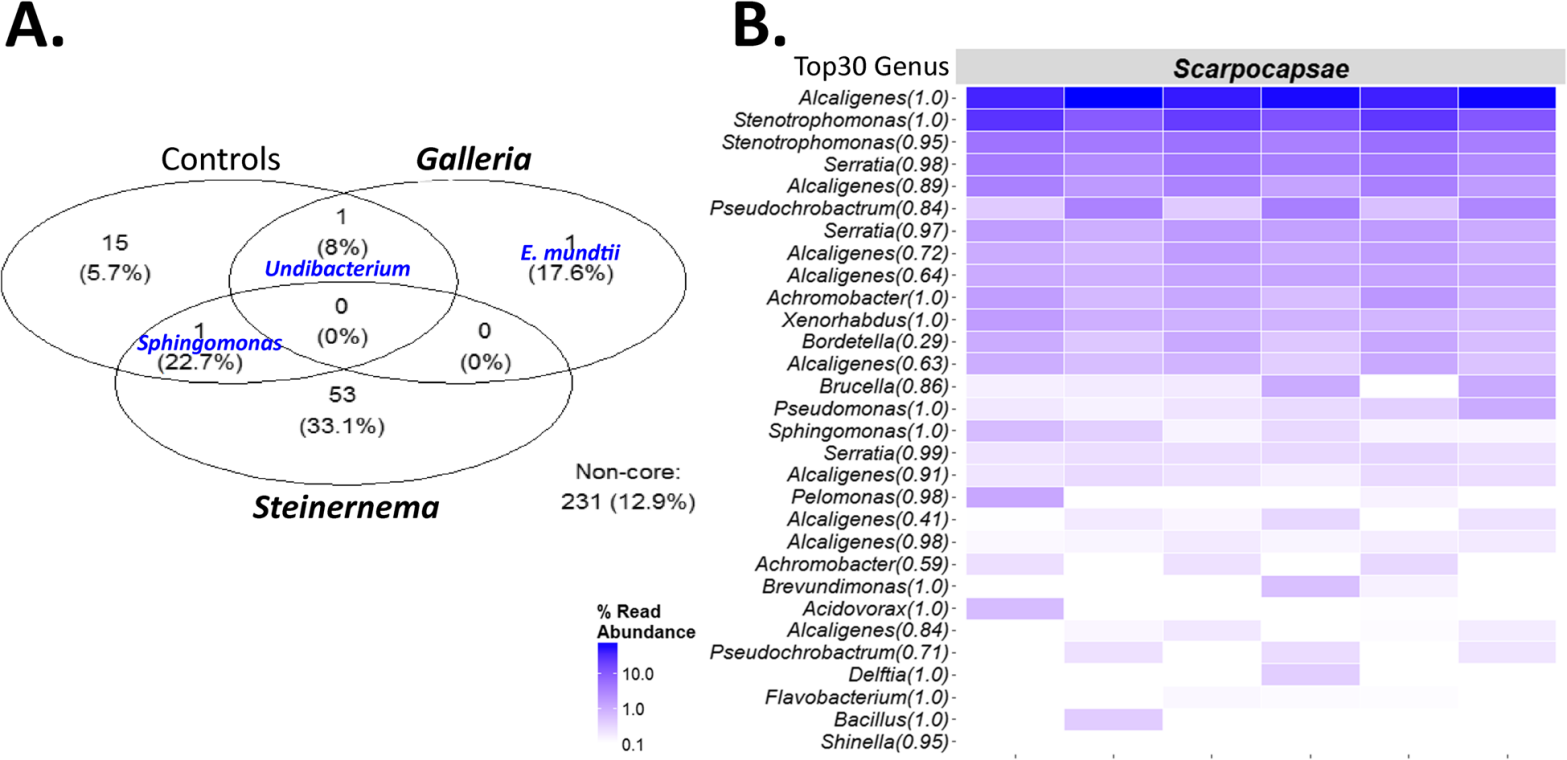
Bacterial communities associated with IJs of *S*. *carpocapsae* SK27, *Galleria mellonella* larvae and control samples. The IJ microbiota was investigated by metabarcoding with the V3V4 region of the 16S rRNA gene. **a** Venn diagram of the OTU detected in of *S*. *carpocapsae* SK27 batch SK27_23_08_16 samples, *G*. *mellonella* samples and control samples (Kitome_QE, Kitome_MN, Tap water and Ringer). OTUs (frequency cut-off > 80% and abundance cut-off > 0.01%) were assigned to genus level. Non-core set embeds OTUs that are not shared by all the control, the *Galleria* or the *Steinernema* replicates. **b** Heatmap showing the composition of the microbiota of *S*. *carpocapsae* SK27 batch SK27_23_08_16 samples. Each column represents a technical replicate. The 30 most abundant OTUs across the samples at the genus level of affiliation (Top30 Genus) are listed on the left. The percentage relative abundance is indicated by the gradient of blue hues.

The community structure observed differed significantly between IJs, *Galleria* and control samples (Additional file 4). The IJ sample and control samples contained mostly Proteobacteria, including many members of the *Burkholderiaceae*. However, only one OTU, affiliated to the *Sphingomonas* genus, was common to both the control and IJ samples (Fig. 2a). As previously reported in different Lepidoptera [20, 39, 40], the *Galleria* samples consisted mainly of Firmicutes, and an OTU affiliated to the genus *Enterococcus* was consistently detected (Additional file 4). No OTU common to the IJ and *Galleria* samples was identified (Fig. 2a). We therefore (i) removed the OTUs affiliated to the genus *Sphingomonas* from subsequent analysis and (ii) concluded that the multiplication of IJs in *Galleria* larvae does not influence the *Steinernema* microbiota. In each replicate of the IJ samples, the OTU identified as *X*. *nematophila*, the *S*. *carpocapsae* symbiont, was one of the 30 most abundant OTUs (Top30), but other OTUs were also present (Fig. 2b), demonstrating the association of a bacterial community much more complex than the symbiont with the IJ samples.

In metabarcoding analysis based on the V3V4 region of the 16S marker, only 57 and 12% of the OTUs could be associated with a genus or species name, respectively (Additional file 5). For refinement of the taxonomic assignment of OTUs, we used the *rpoB* marker, a 435-bp region of the housekeeping *rpoB* gene [41]. The sequencing of *rpoB* marker amplicons generated 96,825 reads (mean = 16,137 reads per sample; number of samples = 6). Rarefaction curves generated on quality-filtered reads indicated that sequencing depth was sufficient (Additional file 2). As previously observed [41], the *rpoB* marker gave a better taxonomic assignment, with 56% and 32% of the OTUs associated with a genus or species name, respectively (Additional file 5). Based on these results, we pursued the two-marker approach.

### Microbial community patterns segregate according to the *Steinernema* species

**Fig. 3:**
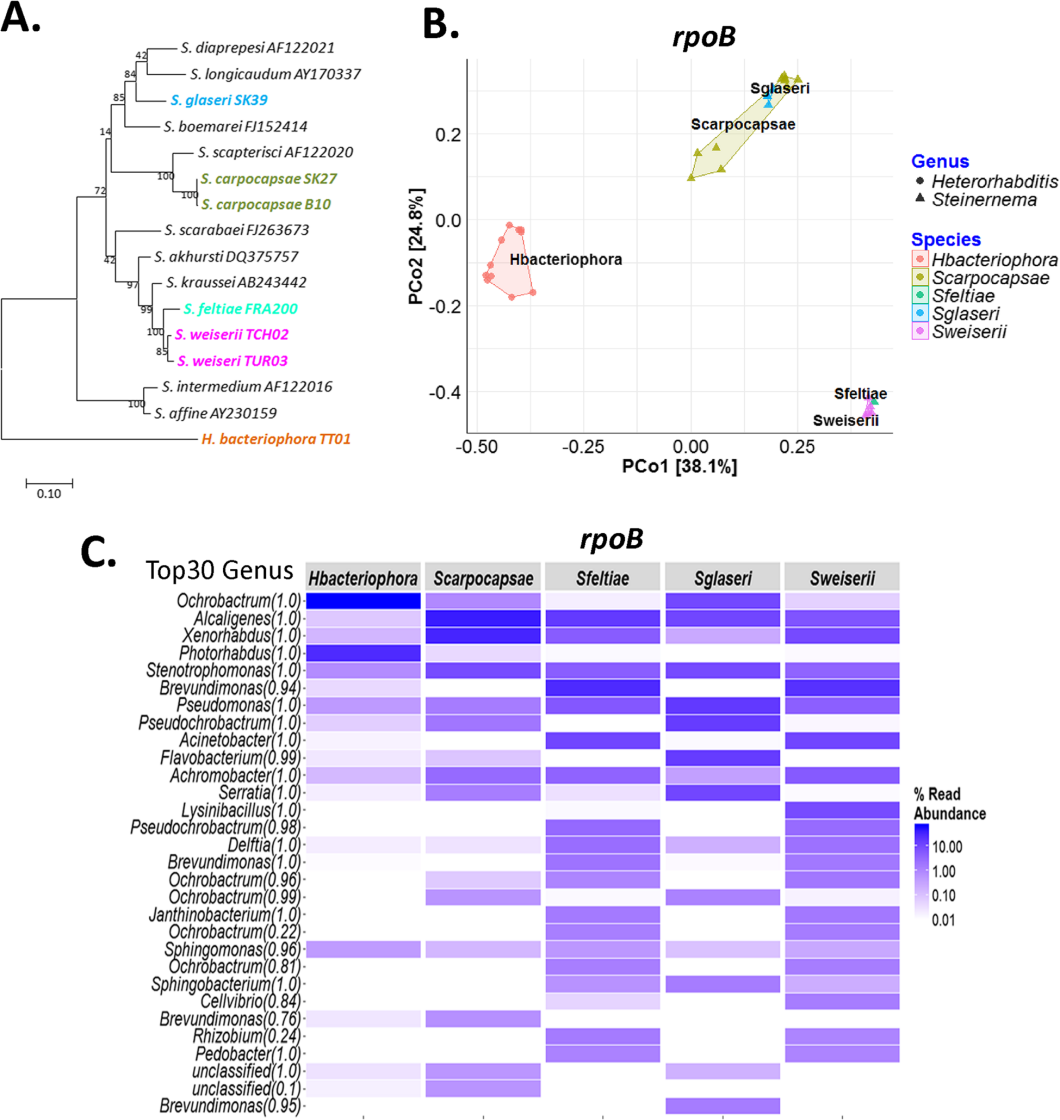
Bacterial communities associated with IJs from different species of *Steinernema*. **a** Simplified phylogeny of *Steinernema*. Phylogenetic relationships between 16 *Steinernema* strains based on a maximum likelihood (ML) analysis of partial internal transcribed spacer (ITS) regions (~ 850 bp). *Heterorhabditis bacteriophora* was used as outgroup. Branch support values (estimated by the aLRT [SH-like] method) are shown at the nodes (percentages of 100 replicates). The branch length scale bar below the phylogenetic tree indicates the number of nucleotide substitutions per site. The accession numbers of the sequences are indicated after the names of the nematodes. The sequences of strains used for metabarcoding studies are shown in colour. **b** Principal co-ordinates analysis (PCoA) based on Bray-Curtis distances for the IJ microbiota. *Heterorhabditis* and *Steinernema* samples are indicated by circles and triangles, respectively. Colours indicate the different *Steinernema* species. Each point represents a technical replicate. The proportion of the variance explained by each axis is shown. The five samples are significantly different (Permanova *rpoB*, Df = 4, *R*^2^ = 0.73, *p* value = 10^−4^). **c** Heatmap showing the microbiota composition of IJ samples. Each column represents an IJ species. The 30 most abundant OTUs across the samples at the genus level of affiliation (Top30 Genus) are listed on the left. The percentage relative abundance is indicated by the gradient of blue hues. For **b** and **c**, IJ microbiota were described by metabarcoding with the 435 bp *rpoB* region. The EPN strains used here belong to the following batches: *S*. *carpocapsae* SK27_23_08_16 and B10_27_04_16; *S*. *weiseri* 583_09_06_15, TCH02_11_08_16 (t1), *S*. *glaseri* SK39_09_06_15; *S*. *feltiae* FRA200_09_06_15 and FRA200_12_08_15 and *H*. *bacteriophora* TT01_22_06_16 and TT01_15_03_16. Three to six technical replicates per batch were performed

Are IJ microbiota composition and richness influenced by the host species? We tested this hypothesis by describing the microbial communities associated with IJ from different *Steinernema* species (Fig. 3a). As an outgroup of the genus *Steinernema* [42], we included *Heterorhabditis bacteriophora* TT01, another entomopathogenic nematode harbouring symbiotic *Photorhabdus luminescens* bacteria. After quality filtering, we were left with a mean of 21,635 sequences per sample (*n* = 45) corresponding to 317 OTUs for the V3V4 marker, and 14,935 sequences per sample (*n* = 34) corresponding to 241 OTUs for the *rpoB* marker. Rarefaction curves confirmed that sequencing coverage was adequate (Additional file 2). Alpha diversity was similar for the two markers (Additional file 3). The community structure found in the various IJs (Fig. 3b and Additional file 6) differed significantly between the outgroup *H*. *bacteriophora* and *Steinernema* samples (Permanova V3V4, Df = 1, *R*^2^ = 0.28, *p* value = 10^−4^; Permanova *rpoB*, Df = 1, *R*^2^ = 0.38, *p* value = 10^−4^), regardless of the marker considered, but also between the *Steinernema* species (Permanova V3V4, Df = 3, *R*^2^ = 0.37, *p* value = 10^−4^; Permanova *rpoB*, Df = 3, *R*^2^ = 0.59, *p* value = 10^−4^). As previously shown for *S*. *carpocapsae* SK27_batch 23_08_16, Proteobacteria were prominent members of the IJ microbiota (Fig. 3c and Additional file 6). Microbial patterns seem to be correlated to the nematode host’s phylogenic tree (Fig. 3a), suggesting co-evolution of the microbiome and its host. Further analyses including a larger number of strains would be necessary to confirm this phylosymbiosis signal.

### A bacterial community is frequently associated with *S*. *carpocapsae* IJs

**Fig. 4:**
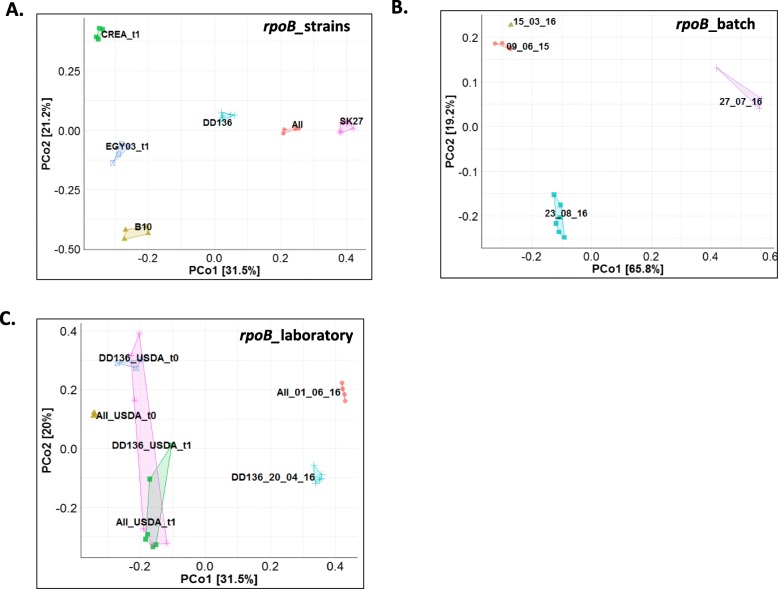
Bacterial communities associated with different samples from the species *S*. *carpocapsae*. Principal co-ordinate analysis (PCoA) based on Bray-Curtis distances for IJ microbiota obtained by metabarcoding with the 435-bp *rpoB* region. Each point represents an individual sample replicate. The different strains, multiplication batches and origins of *S*. *carpocapsae* are indicated by colours and symbols. The proportion of variance explained by each axis is shown. **a** Comparison of six *S*. *carpocapsae* strains. The six samples are significantly different (Permanova, Df = 5, *R*^2^ = 0.87, *p* value = 10^−4^). **b** Comparison of four multiplication batches of *S*. *carpocapsae* SK27. The four samples are significantly different (Permanova, Df = 3, *R*^2^ = 0.75, *p* value = 10^−4^). **c** Comparison of two laboratory origins of *S*. *carpocapsae* DD136 and *S*. *carpocapsae* All (Permanova: DD136 versus All, Df = 1, *R*^2^ = 0.12, *p* value = 10^−4^; DGIMI versus USDA, Df = 1, *R*^2^ = 0.25, *p* value = 10^−4^)

Is there a core community associated with the laboratory-reared *Steinernema* species? We explored this question by focusing on *S*. *carpocapsae* species and comparing the community composition of IJs from six *S*. *carpocapsae* strains reared in the laboratory for several years (Additional file 1). SK27 (from four different multiplication batches), B10, EGY03, CREA, All_DGIMI and DD136_DGIMI were all maintained in our laboratory (DGIMI, Montpellier, France). All_USDA and DD136_USDA were maintained at the USDA-ARS, Fruit and Tree Nut Research Unit at Byron, Georgia, USA. The community structure of the various *S*. *carpocapsae* samples (Fig. 4 and Additional file 7) revealed that strain, multiplication batch and storage laboratory accounted for a significant proportion of the variance of IJ microbiota (Permanova *rpoB*_strains, Df = 5, *R*^2^ = 0.87, *p* value = 10^−4^; Permanova V3V4_strains, Df = 5, *R*^2^ = 0.75, *p* value = 10^−4^; Permanova *rpoB*_batch, Df = 3, *R*^2^ = 0.75, *p* value = 10^−4^; Permanova V3V4_batch, Df = 3, *R*^2^ = 0.74, *p* value = 3.10^−4^; Permanova *rpoB*_laboratory, Df = 1, *R*^2^ = 0.25, *p* value = 10^−4^; Permanova V3V4_laboratory, Df = 1, *R*^2^ = 0.28, *p* value = 10^−4^). Likewise, when we compared the IJ microbiota of the American strains on reception at our laboratory (All_USDA_t0 and DD136 USDA_t0) and after just one round of multiplication in *Galleria* larvae in our laboratory (All_USDA_t1 and DD136 USDA_t1), we found that a quarter of the variance depended on batch (t0 versus t1 in Fig. 4c and Additional file 7) (Permanova V3V4, t0 versus t1, Df = 1, *R*^2^ = 0.27, *p* value = 10^−4^; Permanova *rpoB*, t0 versus t1, Df = 1, *R*^2^ = 0.24, *p* value = 2.10^−4^). Our results thus suggest that the rearing and storage of IJs in the laboratory lead to a drift.

**Fig. 5:**
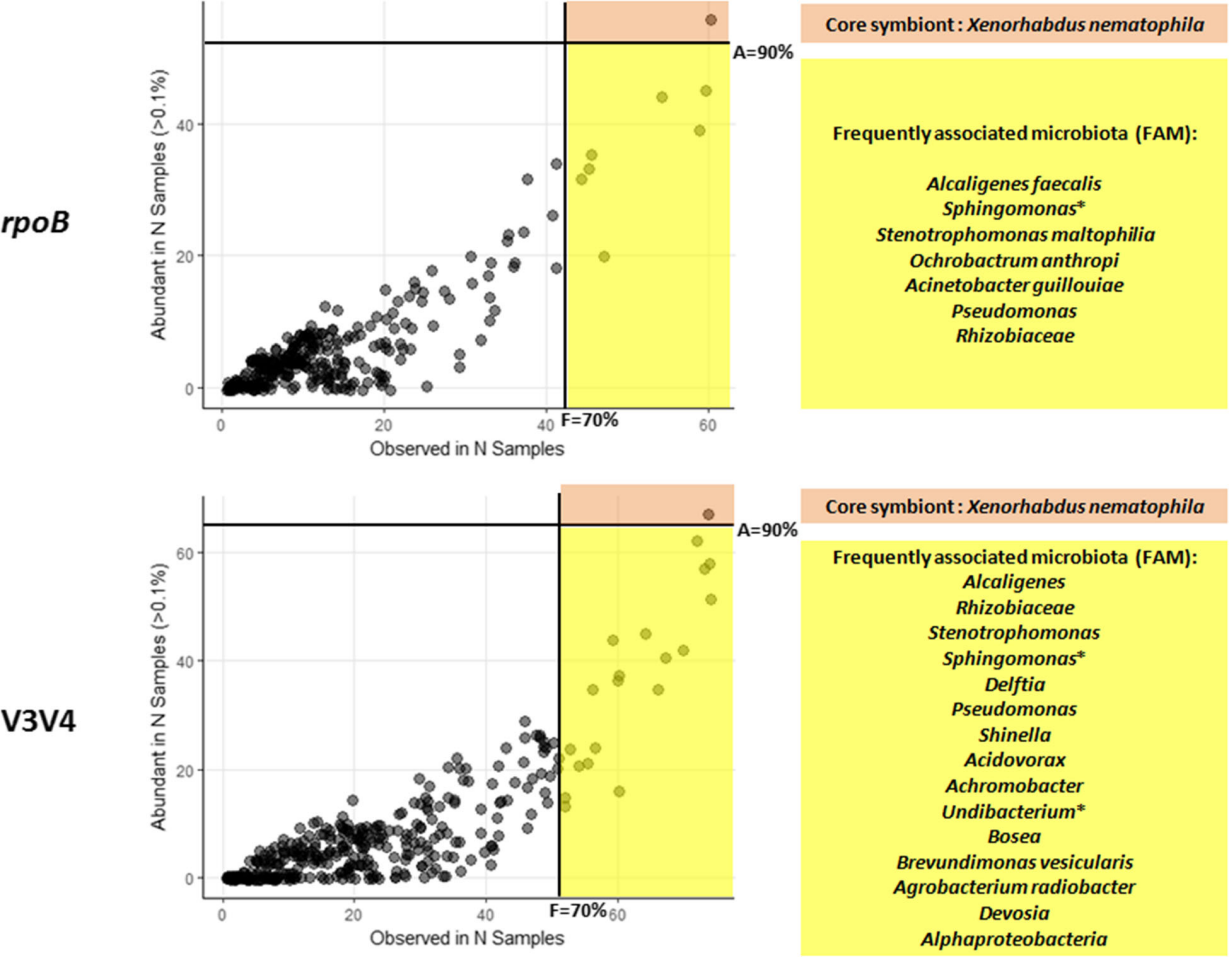
Occurrence (*x* axis) and frequency of high abundance (*y* axis) of OTUs associated with laboratory-reared *S*. *carpocapsae* IJs. Abundant OTUs with read numbers accounting for more than 0.1% of the total reads for the sample were plotted. The darker the dot, the more OTUs with these characteristics are found at the position concerned. Among the OTUs with high occurrence rates (present in more than 70% of the samples), the yellow and orange areas delimit two groups. Orange group: frequency of high abundance > 90% (core symbiont). Yellow group: frequency of high abundance < 90% (frequently associated microbiota or FAM). OTU identity is indicated to the right of the graph (bootstrap confidence ≥ 0.9). *OTU present in the control samples

Despite these variations and regardless of the marker used, we detected recurrent OTUs in addition to the cognate symbiont *X*. *nematophila* among the 30 most abundant OTUs of the whole set of *S*. *carpocapsae* samples (Additional file 8). For each abundant OTU (read number > 0.1% of the sample read), we represented the frequency of abundance as a function of frequency (Fig. 5). For OTUs with a high occurrence (present in at least 70% of the samples), we identified two groups. In the first group, the *X*. *nematophila* OTU was always abundant (present in at least 90% of the samples), confirming its status as the core symbiont of *S*. *carpocapsae* (Fig. 5). In the second group, the OTUs were abundant in less than 90% of the samples. We named this set of OTUs the “frequently-associated microbiota” (FAM). After removal of the OTU found in the controls (*Sphingomonas*), the FAM common to the two markers encompassed the genera *Alcaligenes*, *Stenotrophomonas*, *Pseudomonas* and the *Rhizobiaceae* family (Fig. 5).

We checked for the FAM of laboratory-reared *S*. *carpocapsae* in the IJs of *S*. *carpocapsae* GRAB recently sampled from soils by the ex situ *Galleria* trap method (Fig. 1a) in an apple orchard in November 2017 (Additional file 1). Metabarcoding analysis with both V3V4 and the *rpoB* markers was performed on GRAB IJs shortly after trapping (only one round of multiplication in *Galleria* larvae). Bacterial diversity was higher than that in nematode lineages multiplied for long periods in the laboratory, but both markers identified the core symbiont *Xenorhabdus nematophila* and the FAM members *Stenotrophomonas*, *Pseudomonas* and *Rhizobiaceae*, but not *Alcaligenes*, suggesting that the identification of this last taxon might be due to a bias caused by laboratory rearing (Additional file 9).

### Bacteria associated with *S*. *carpocapsae* IJs are cultivable

**Fig. 6:**
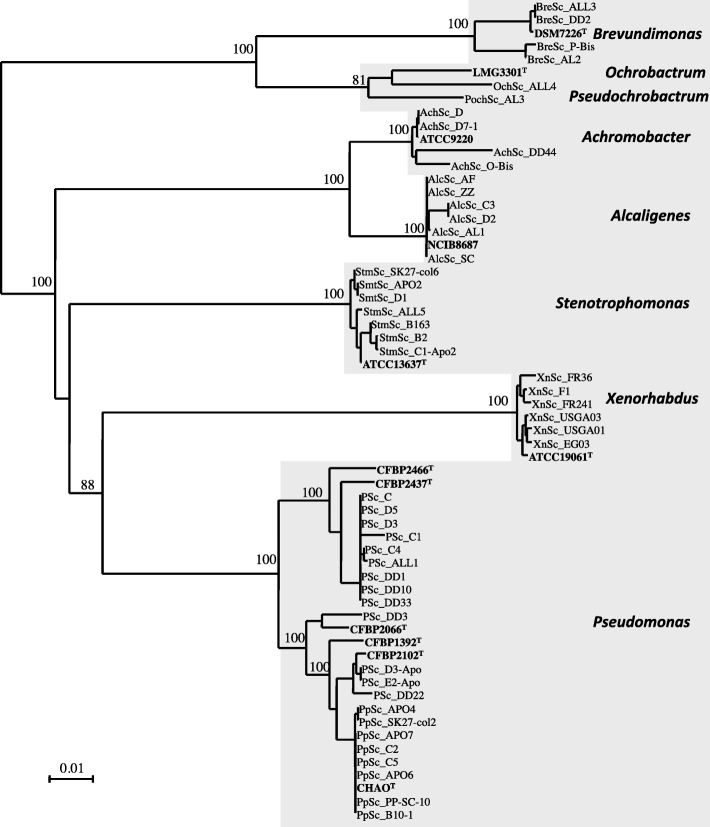
Distance phylogenetic tree of 62 bacterial isolates from *S*. *carpocapsae* IJs. The phylogenetic tree of taxa isolated from *S*. *carpocapsae* IJs was constructed with the 16S rRNA gene sequences (1377 nucleotides), with the Kimura two-parameter model [43] and the neighbour-joining method [44] included in SeaView 4.7 software. Bootstrap values (percentages of 1000 replicates) of more than 90% are shown at the nodes. Twelve type strains (in bold) of the *Xenorhabdus*, *Pseudomonas*, *Stenotrophomonas*, *Alcaligenes*, *Ochrobactrum*, *Pseudochrobactrum*, *Achromobacter* and *Brevundimonas* genera were added. The bar represents 1% sequence divergence

We performed a large survey of the isolation on nutrient-rich culture media of bacteria from ground *S*. *carpocapsae* IJs or from the content of *G*. *mellonella* cadavers infested with *S*. *carpocasae* IJ. We isolated only Gram-negative taxa. We regularly sampled isolates from the following eight genera: *Brevundimonas*, *Ochrobactrum*, *Pseudochrobactrum*, *Achromobacter*, *Alcaligenes*, *Stenotrophomonas*, *Xenorhabdus* and *Pseudomonas* (Additional file 1 and Fig. 6). These taxonomic groups matched with the OTUs identified by metabarcoding in 70% of the *S*. *carpocapsae* samples with one of the two markers (Fig. 5). Bacterial isolation on rich agar medium cannot be used for the quantification of taxa. However, for some samples, *Xenorhabdus* colonies were less abundant on NBTA plates than the other taxa, such as *Stenotrophomonas*, *Alcaligenes* and *Pseudomonas*. Culture-dependent approaches thus validate the *S*. *carpocapsae* FAM identified by the molecular approach.

### Investigation of FAM functions potentially involved in the parasitic lifecycle of *S*. *carpocapsae*

The ability of *S*. *carpocapsae* to kill insects is thought to be due to the presence of the symbiont *X*. *nematophila* [18]. However, other bacteria associated with the *S*. *carpocapsae* IJs might also contribute to entomopathogenicity. We assessed the pathogenicity of several of the bacteria frequently associated with the *S*. *carpocapsae* IJs. When 10^2^–10^3^ CFU/mL were injected into larvae of the lepidopteran insect *Spodoptera littoralis*, the highly pathogenic symbiont *Xenorhabdus nematophila* XnSc_F1 and *Pseudomonas protegens* strain PpSc_PP-SC-10, both isolated from *S*. *carpocapsae* SK27, took similar time to kill 50% of the larvae (LT_50_): 27 and 29 h, respectively (*S*. *carpocapsae* microbiota in Table 1). The larvae were not killed by other bacteria from the *S*. *carpocapsae* FAM (*S*. *maltophilia*, *Alcaligenes faecalis*, *Pseudochrobactrum*, *Ochrobactrum*), even at doses of 10^5^–10^6^ CFU/mL.

**Table 1:** Lethal time 50 values (LT_50_) for *Spodoptera littoralis* following the injection of bacterial strains into the haemocoel of last-instar larvae (incubated at 23 °C)

	Strain	Dose range	LT50 (h)^a^
*S*. *carpocapsae* microbiota	*Xenorhabdus nematophila* XnSc_F1	10^2^–10^3^	27
	*Pseudomonas protegens* PpSc_PP-SC-10	10^2^–10^3^	29
	*Stenotrophomonas maltophilia* StmSc_ALL5	10^5^–10^6^	No mortality
	*Alcaligenes faecalis* AlcfSc_SC	10^5^–10^6^	No mortality
	*Pseudochrobactrum* PochSc_AL3	10^5^–10^6^	No mortality
	*Ochrobactrum sp* OchSc_ALL4	10^5^–10^6^	No mortality
*S*. *glaseri* SK39 microbiota	*Xenorhabdus poinarii* XpSg_G6	10^2^–10^3^	No mortality
	*Pseudomonas protegens* PpSg_SG6 Apo	10^2^–10^3^	28
	*Pseudomonas chlororaphis* PcSg_SK39 ApoA	10^2^–10^3^	19
	*Stenotrophomonas maltophilia* StmSg_SK39-2	10^5^–10^6^	No mortality
*S*. *weiseri* 583 microbiota	*Xenorhabdus bovienii* XbSw_CS03	10^5^–10^6^	No mortality
	*Pseudomonas protegens* PpSw_SW4	10^2^–10^3^	20
	*Pseudomonas protegens* PpSw_TCH07 2-2	10^2^–10^3^	26
	*Stenotrophomonas maltophilia* StmSw_SW1	10^5^–10^6^	No mortality
	*Stenotrophomonas maltophilia* StmSw_TCH07 2-3	10^5^–10^6^	No mortality
	*Ochrobactrum anthropi* OchaSw_SW2	10^5^–10^6^	No mortality
*H*. *bacteriophora* microbiota	*Photorhabdus luminescens laumondii* PholHb_TT01	10^3^	32
	*Ochrobactrum anthropi* OchaHb_B3	10^5^–10^6^	No mortality
Other *Pseudomonas* strains	*Pseudomonas protegens* CHAO^T^	10^2^–10^3^	28
	*Pseudomonas chlororaphis* CFBP 2132^T^	10^2^–10^3^	19
Negative control for pathogenicity	*Escherichia coli* CIP 7624	10^5^–10^6^	No mortality

^a^Mortality was recorded over 72 h following the intrahaemocoel injection of 10^2^–10^3^ or 10^5^–10^6^ washed bacterial cells/larva. LT_50_ was calculated on 20 larvae

It is also generally asserted that *X*. *nematophila* outcompetes other bacterial strains or species during its lifecycle, particularly during the infestation phase of insect cadavers [20, 45]. We therefore monitored the in vitro antibiosis of the *X*. *nematophila* XnSc_F1 strain against the cultivable bacterial members of the *S*. *carpocapsae* microbiota. Only two of the six bacteria isolated (*Alcaligenes faecalis* Alcf_SC and *Ochrobactrum* sp. OchSc_ALL4) displayed slight susceptibility to the production of antimicrobials by *X*. *nematophila* XnSc_F1 (Additional file 10). By contrast, five of these six bacteria were susceptible to the antimicrobial production of *P*. *protegens* PpSc_PP-SC-10 (Additional file 10).

### *P*. *protegens* and *P*. *chlororaphis* are important functional members of the *Steinernema* FAM

**Fig. 7:**
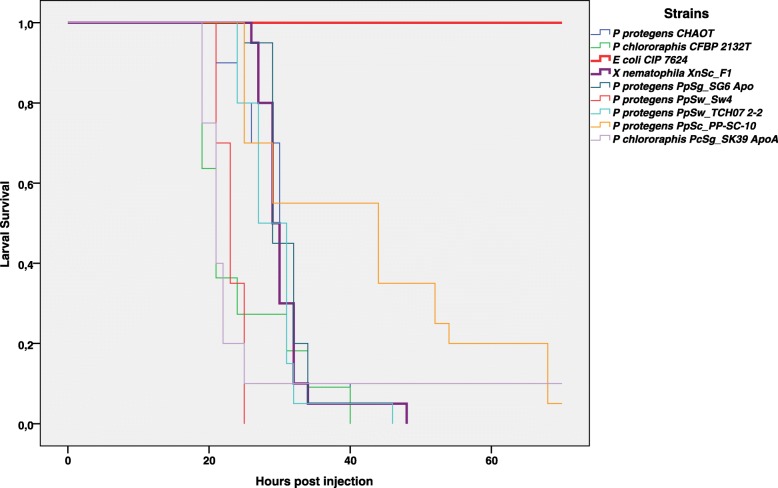
Survival curves of the insect *Spodoptera littoralis* after the direct injection of IJ-associated *Pseudomonas protegens* and *Pseudomonas chlororhaphis*. IJ-associated *Pseudomonas*: *P*. *protegens* strains PPSg_SG6 APO, PpSw_SW4, PpSw_TCH07 2-2, PpSc_PP-SC-10 and *P*. *chlororaphis* strain PcSg_SK39 ApoA. Rhizosperic strains: *P*. *protegens* CHAO^T^, *P*. *chlororaphis* CFBP2132^T^. Positive controls: *X*. *nematophila* strain XnSc_F1 (bold purple curve). Negative control (bold red curve): *E*. *coli* CIP7624. We injected 10^2^ or 10^3^ bacterial cells in the exponential growth phase and injected each of 20 last-instar larvae

During this study, we also recovered a panel of bacterial strains from IJs of three other entomopathogenic nematodes: *S*. *glaseri* SK39 and *S*. *weiseri* 583 that carried the symbionts *X*. *poinarii* XpSg_G6 and *X*. *bovienii* XbSw_CS03, respectively, and *H*. *bacteriophora* TT01 that carried the symbiont the symbiont *P*. *luminescens* PholHb_TT01. *X*. *poinarii* XpSg_G6 and *X*. *bovienii* XbSw_CS03 were previously reported to be attenuated in virulence [46–48], while *P*. *luminescens* PholHb_TT01 is highly virulent [49]. As in *S*. *carpocapase*, bacterial isolates from *Steinernema* IJs were generally more susceptible to the antimicrobials produced by *P*. *protegens or P*. *chlororaphis* than those produced by *Xenorhabdus* (Additional file 10). We assessed the pathogenicity of these bacterial isolates by injection in *S*. *littoralis* larvae (Table 1). For isolates from *S*. *glaseri* SK39 and *S*. *weiseri* 583, only *P*. *protegens* and *Pseudomonas chlororaphis* strains were able to kill the insect larvae (Table 1). They had LT_50_ values between 19 and 29 h, in the same range as the values obtained with reference strains isolated from rhizosphere environments, *P*. *protegens* CHAO^T^ and *P*. *chlororaphis* CFBP2132^T^ [50–53] (Table 1 and Fig. 7).

## Discussion

This exploration of the monoxenic paradigm for the *Xenorhabdus*-*Steinernema* symbiotic interaction provided us with an opportunity to explore, for the first time, the bacterial microbiota associated with entomopathogenic nematodes of the genus *Steinernema*. To this end, we used two approaches to analyse a large panel of *Steinernema* infective stages. We first molecularly characterised the microbiota of four *Steinernema* species with two markers, the V3V4 region of the 16S rRNA gene and a 435 bp region of the *rpoB* gene, because multigene approaches have been shown to improve the reliability of bacterial community description in ecosystems of low complexity [41]. We also characterised the *Steinernema* microbiota by culture-based methods. This approach differed from that used in previous culture-dependent studies in the considerable effort made to isolate a large set of colonies (71, selected from more than 350 isolates, in this study) and to perform molecular taxonomic characterization on these colonies.

In the previous culture-dependent studies, questions were frequently raised about the source of the other bacteria than the symbiont found in *Steinernema* IJs. The authors often concluded that these taxa were contaminants [26], except for *Ochrobactrum* strains that were considered to be involved in a dixenic association with *Photorhabdus* in the EPN *Heterorhabditis* [54]. Indeed, as the isolated taxa are frequently detected in the healthy insect gut, in soils and in the haemolymph of insects infected with *Steinernema* [20, 32, 55, 56], it is generally assumed that the non-symbiotic bacteria randomly “hitchhike” in IJ vectors via the cuticle or intercuticular space and are introduced in the insect haemocoel during IJ penetration [27, 29].

Here, we clearly described the microbiota associated with *Steinernema carpocapsae* reared in laboratory as composed of the core symbiont, *Xenorhabdus*, and a frequently associated microbiota (FAM) consisting of cultivable Proteobacteria also identified in IJs freshly isolated from soil, their natural habitat. The only exception was *Alcaligenes*, which was detected only in laboratory-reared IJs. A large number of reads attributed to *Alcaligenes* was generally correlated with a high level of abnormal morphology (Ogier and Pagès, unpublished data). Poinar also described morphological and behavioural abnormalities in the nematode *Heterorhabditis heliothis* when IJs developed in presence of *Alcaligenes faecalis* [57], suggesting that a high abundance of this genus reflects drift due to laboratory rearing in batches of sick nematodes.

The Proteobacteria of the FAM are frequently encountered in the microbiota of organisms or compartments associated with soils. For instance, we detected taxa previously identified by culture-independent studies on other parasitic or soil nematodes. *Pseudomonas* has been detected and isolated from soil-dwelling forms of the nematode *Pristionchus*, a parasite of scarab beetles [58]. *Stenotrophomonas*, *Ochrobactrum* and *Pseudomonas* have repeatedly been identified in the soil-dwelling *Acrobeloides maximus* and *Caenorhabditis elegans* nematodes [36–38]. Likewise, Proteobacteria is the most abundant phylum in bacterial communities associated with plant roots [59, 60] as well as in plant-covered soils, such as the rhizosphere or litterfall [61, 62].

The composition of the *Steinernema* microbiota seems to be shaped by the ecological niche, but the question remains as to whether the establishment of the association between the bacterial community and the nematode is due to the chance alone or to specific mechanisms of association. In this study, we provide an array of arguments in favour of specific association with a larger microbiota than the core symbiont alone. First, Proteobacteria frequently encountered in the microbiota of organisms or compartments associated with soils were identified in nematodes that had been reared in the laboratory for years, suggesting that the nematode-microbiota association is robust enough to be sustained in non-natural (laboratory rearing in the non-natural host *Galleria*) and very different (France and USA) environments. Second, microbiota profiles distinguished between the genera *Heterorhabditis* and *Steinernema*, and also between the four *Steinernema* species studied here. Further studies are required to determine whether IJ microbiota composition actually reflects the entomopathogenic species phylogeny, as demonstrated for the gut bacteria of the wasp *Nasonia* [63], for coral-associated bacteria [64] and for insect viromes [65]. However, these preliminary data support the notion that some members of the *Steinernema* microbiota may be vertically (or pseudovertically) transmitted to the progeny, as reported for the core symbiont *Xenorhabdus* [66]. This would not rule out the possibility of hybrid transmission, with more “accessory” microbiota members transmitted horizontally, as reported for a nematode colonising the vertebrate gastrointestinal tract, *Haemonchus contortus* [13].

The description of an IJ-associated microbiota for *S*. *carpocapsae* and other *Steinernema* species is important because it improves our understanding of the ecological functioning of EPN. Indeed, the killing efficiency of nematodes was long thought to depend principally on the action of *Xenorhabdus* [16–18]. However, several studies have cast doubt on this view, because some entomopathogenic pairs have been found to have attenuated virulence or to be non-virulent when injected alone into insects [46–48, 67, 68]. These previous findings and the results presented here call into question the notion of the “pathobiome” in the case of the EPN ecology. *Pseudomonas protegens* and *Pseudomonas chlororaphis*, two rhizospheric and entomopathogenic species from this genus [69], are two good candidate members of the IJ pathobiome. An entomopathogenic *Pseudomonas fluorescens* strain has already been isolated by Lysenko and Weiser from *S*. *carpocapsae* IJs [28]. At the time *P*. *fluorescens* taxonomy was far from fully elucidated, but we assume that the authors actually identified a strain of *P*. *protegens* or *P*. *chlororaphis*.

The EPN lifecycle is not restricted to the killing of the insect. The nematode completes its sexual reproduction within the killed insect, then re-associates specifically with the symbiont, and possibly other members of its microbiota, after which it survives in the soils [16–18]. If *P*. *protegens* or *P*. *chlororaphis* belong to the IJ-associated microbiota, how do they interact with the other members of the IJ microbiota? An assessment of in vitro antimicrobial activity on agar plates showed that the *Xenorhabdus* strains have weak antimicrobial activity against bacterial members of *Steinernema* microbiota, whereas *P*. *protegens* and *P*. *chlororaphis* strains displayed higher levels of antimicrobial activity against both *Xenorhabdus* strains and other bacterial members of the *Steinernema* microbiota. If antibiotic activity measured in vitro accurately reflects the antibiotic activity in vivo, it suggests that antimicrobial production from species other than *Xenorhabdus* may be involved in in vivo interspecies competition. These data also suggest that the various members of the *Steinernema* microbiota might be subject to spatial compartmentation.

## Conclusions

Finally, increasing numbers of scientists in the community working on microbial ecology are stressing the importance of working on natural microbial communities of low genotypic, functional or environmental complexity for the purposes of experimentation and theoretical modelling [70]. We are convinced that the microbial communities associated with EPNs meet this need perfectly. Three main questions could be explored in future studies of EPN models. Where are the members of this frequently IJ-associated microbiota located? How are these bacteria transmitted between EPN generations? What impact does each member of the microbiota have on entomopathogenic nematode fitness, survival and the capacity for adaptation in changing environments (soils, insects, etc.)?

## Methods

### Biological material

All the entomopathogenic nematodes (EPN) strains used here are listed in Additional file 1. Bacteria isolated from EPNs or EPN-infested *Galleria* cadavers and used for pathology and/or antibiosis assays are listed in Additional file 11. Bacteria used for the construction of the phylogenetic distance tree are listed in Additional file 12. *Galleria mellonella* (Lepidoptera: Pyralidae) insect larvae were reared at 28 °C in the dark, on honey and pollen. *Spodoptera littoralis* (Lepidoptera: Noctuidae) insect larvae were reared on an artificial diet [71] at 23 ± 1 °C, with a photoperiod of 16 h of light and 8 h of darkness (L16:D8) and relative humidity (RH) of 40 ± 5%.

### Isolation, multiplication and storage of EPNs

EPN were isolated by ex situ *Galleria* trap (Fig. 1a) as previously described [72]. We used laboratory-reared EPN stocks, except *S*. *carpocapsae* GRAB, which was isolated in November 2017 from an apple orchard near of Avignon, France (43°54′20.4″ N 4°53′09.0″ E). Briefly, soil samples were collected at a depth of 0–20 cm (temperature 5.5 to 8.2 °C; RH 15.2 to 21.4%) and placed in 1-L plastic containers containing five last-instar *Galleria* larvae, which were then stored in the dark at 18 °C for seven days. The *Galleria* cadavers were placed on White traps (Fig. 1a). IJs emerging from cadavers and their cognate symbiont were identified molecularly (see below for details) as *S*. *carpocapsae* (100% identity for the ITS1-2 region) and *X*. *nematophila* (100% identity for the 16S rRNA), respectively. IJs were stored in Ringer’s solution (Merck). Laboratory-reared EPN stocks were multiplied every six months by infestation of last instar of *Galleria* as previously described [73]. Briefly, the EPNs were placed in contact with the *Galleria* larvae in Petri dishes. When the *Galleria* larvae died, the cadavers were placed on a White trap (Fig. 1b). Emerging IJs of *Steinernema* and *Heterorhabditis* species were stored in Ringer’s solution at 9 °C and 15 °C, respectively.

### Isolation, culture and storage of bacteria isolated from EPNs

The bacteria were isolated by the hanging drop technique [23] (for *Xenorhabdus* and *Photorhabdus* only), from content of *G*. *mellonella* cadavers after IJ infestation or by crushing IJs. For the crushing method, we placed 20 IJs in a 1.5-mL Eppendorf tube containing 200 μL of LB broth and three 3-mm glass beads and subjected them to three cycles of grinding (1 min, 30 Hz, followed by 1 min without agitation) in a TissueLyser II apparatus (Qiagen, France). Bacteria were isolated on nutrient agar (Difco) plates or on nutrient bromothymol blue agar (NBTA) plates [74] and incubated at 28 °C for 48 h. Bacteria were also routinely grown at 28 °C in Luria-Bertani (LB) broth. The bacteria were stored at − 80 °C with 16% glycerol (v/v).

### Molecular identification of EPNs and isolated bacteria

*Steinernema* species were identified by amplifying and sequencing 850 bp of the ITS1-2 region of the ribosomal DNA sequence as previously described [75], with the following primers, ITS_jc_F: 5′-GGA-CTG-AGC-TGT-TTC-GAG-A-3′ and ITS_jc_R: 5′-TAC-TGA-TAT-GCT-TAA-GTTCAG-CG-3′. Bacterial isolates were identified as previously described [76] by amplifying and sequencing a near full-length 16S rRNA gene (1372 bases). PCR amplifications were conducted in a Bio-Rad thermocycler (Bio-Rad, USA) and sequencing was performed by Eurofins, Germany.

### Pathogenicity assays

Bacterial pathogenicity was assessed by injection into *S*. *littoralis*, as previously described [77]. Briefly, bacterial cultures in LB broth (#OD = 0.8) were diluted in the culture medium and 20 μL of the resulting bacterial suspension, containing 10^3^ to 10^6^ colony-forming units (CFUs), was injected into the haemolymph of 20 fifth-instar larvae of *S*. *littoralis*. The number of bacterial cells injected into the larvae was determined by plating on nutrient agar and counting the CFUs. After the bacterial injection, the insect larvae were incubated at 23 °C and mortality was monitored for up to 72 h. The pathogenicity of bacterial isolates was determined by measuring the time required for 50% of the insect larvae to be killed (LT50).

### Antimicrobial activity

The antimicrobial activity of the *Xenorhabdus*, *Pseudomonas protegens* and *Pseudomonas chlororaphis* strains against other members of the EPN microbiota was assessed in vitro as previously described [74]. Briefly, 5 μL of an exponential growing culture of the producer strain was spotted onto Mueller-Hinton agar plates (Biokar). The producer strain was grown at 28 °C for 48 h and killed by exposure to chloroform for 30 min, followed by air drying for 15 min. Indicator strains were diluted (1.5% overnight culture) in 10 mL of top agar (LB broth; 0.7% agar) and poured over the producer strain plates to form an overlay. Plates were incubated for 24 h at 28 °C for all indicator strains and at 37 °C for *Micrococcus luteus*, which was used as a positive control. The diameter of the zone of inhibition was measured in millimetres. The assays were performed three times.

### DNA extractions from IJ

We sampled 5000 IJs from a storage batch (Fig. 1b). In preliminary protocol tests, we treated nematodes with bleach solutions of different concentrations (from 2% to 10%) as previously performed [19, 32], but microscope observation showed that it resulted in the destruction of the external cuticle in many IJs. To avoid the alteration of the IJ integrity and consequently the bias in their microbiota composition, we decided to minimize contamination with micro-organisms from the body surface by rinsing the IJs thoroughly with tap water, on a filter. The washed IJs were recovered from the filter with a sterile pipette, transferred to 10 mL of sterile ultrapure water and immediately frozen at − 80 °C for future use. We compared several different DNA preparation methods, because EPNs are resistant to lysis because of their double cuticle. DNA recovery rates were highest for mechanical grinding of IJs with three 3-mm glass beads (three cycles of 30 Hz for 2 min, followed by 1 min without agitation) in a TissueLyser II apparatus (Qiagen, France) followed by DNA extraction with the Quick Extract kit from Epi-centre, USA (QE method) or with the Tissue extraction kit from Macherey-Nagel, Hoerdt, France (MN method). Briefly, for the QE method, frozen samples were rapidly thawed, heated at 80 °C for 20 min and centrifuged (3.000×*g*, 10 min) to collect the IJs as a pellet. The IJ pellet was placed in 200 μL of QE lysis buffer and ground. We then added 2 μL of Ready-Lyse Lysozyme Solution (Epi-centre, USA) and incubated the samples at room temperature until the solution cleared (48 to 72 h). Samples were again heated at 80 °C for 20 min, and IJs lysis was checked under a light microscope. When lysis was complete, the sample was treated with 20 μL of RNaseA 20 mg/ml (Invitrogen PureLinkTM RNaseA, France). It was then subjected to phenol-chloroform extraction followed by chloroform extraction alone. DNA was precipitated in 70% ethanol, resuspended in 50 μl ultrapure water and stored at − 20 °C. The MN method was performed according to the kit manufacturer’s instructions, except that the column step was replaced by phenol-chloroform and chloroform extractions, as described above. We assessed contaminant bacterial DNA levels, by generating several control DNA preparations: in ultrapure water with the QE method (Kitome_QE control), in ultrapure water with the MN method (Kitome_MN control), in the tap water used during the IJ washing step, with the QE method (Tap water control) and in the Ringer medium used for IJ storage, with the QE method (Ringer control). For each sample, three to six technical replicates were prepared.

### DNA extraction from *G*. *mellonella* larvae

As IJ multiply within *Galleria* larvae, we also extracted DNA from insect larvae. Three *G*. *mellonella* larvae were surface-sterilised with 70% (vol/vol) ethanol, were placed in a pot with a 2.5 mm tungsten bead and 1.5 mL PBS buffer and were crushed by three grinding cycles (1 min at 30 Hz followed by 1 min without agitation) in the TissueLyzer II apparatus. The homogenate was centrifuged (400×*g*, 2 min) to remove larval debris and the supernatant was immediately frozen at − 80 °C until use. Samples were rapidly thawed, heated at 80 °C for 20 min and split into five fractions. The QE method (see the procedure for IJs described above) was used for the preparation of independent DNA extracts from each technical replicate.

### Library preparation and sequencing

Amplicon libraries were constructed following two rounds of PCR amplification. The first amplification step was performed on 10 to 100 ng of DNA, with iProof^TM^ DNA Polymerase (Bio-Rad), in a Bio-Rad thermocycler. The hyper variable V3V4 region of the 16S rRNA gene was targeted with the universal primers F343 (5′-CTTTCCCTACACGACGCTCTTCCGATCTTA**CGGRAGGCAGCAG**-3′) and R784 (5′-GGAGTTCAGACGTGTGCTCTTCCGATCTTA**CCAGGGTATCTAATCCT**-3′), and the *rpoB* fragment was targeted with the primers Univ_rpoB_F_deg (5′-CTTTCCCTACACGACGCTCTTCCGATCT**GGYTWYGAAGTNCGHGACGTDCA**-3′) and Univ_rpoB_R_deg (5′-GGAGTTCAGACGTGTGCTCTTCCGATCT**TGACGYTGCATGTTBGMRCCCATMA**-3′) [41]. The Illumina adapters are indicated in non-bold characters. We performed 30 amplification cycles with annealing temperatures of 65 °C and 60 °C for the V3-V4 region (~ 460 bases), and the *rpoB* region (~ 435 bp), respectively. Single multiplexing was performed at the Genomic and Transcriptomic Platform (INRA, Toulouse, France), with 6-bp index sequences, which were added to the reverse primers during a second 12-cycle PCR. Amplicon libraries were sequenced with Illumina MiSeq technology (MiSeq Reagent Kit v2) according to the manufacturer’s instructions. FastQ files were generated at the end of the run. The program FastQC [78] was used for quality control checks on raw sequence data and the quality of the run was internally checked by adding 30% of PhiX sequences at 12.5pM. Each pair-end sequence was assigned to its sample with the help of the previously integrated index, and pair-end reads were assembled with FLASH [79]. Unassembled reads were discarded.

### Sequence data processing and analyses

The sequencing reads obtained were processed according to the FROGs pipeline [80]. A pre-processing tool was used to remove sequences that did not contain both primers, to trim the primers and to remove all sequences containing an ambiguous base. Sequence clustering was performed with the Swarm algorithm [81]. Chimeric sequences were detected with the VSEARCH algorithm, by the de novo UCHIME method [82, 83] and were removed. A filtering tool was used to remove clusters, which had a read number abundance of less than 0.005 % of all reads. Sequences were assigned with RDP Classifier [84] and the 16S rRNA database Silva [85] for V3V4 reads. For sequence assignment with the *rpoB* marker, we constructed a reference database including 45,000 sequences; this database is available from the FROGs website (http://frogs.toulouse.inra.fr/).

### Bacterial community and statistical analyses

OTU diversity analysis and statistical analyses were carried out with the R packages Phyloseq [86], Vegan [87] and Ampvis 2 (https://github.com/MadsAlbertsen/ampvis2). In brief, Phyloseq was used for rarefaction curves, α-diversity indices and multivariate PERMANOVA. Ampvis 2 was used for Bray-Curtis distance matrices (square root-transformed OTU abundance data), principal co-ordinate analyses (unconstrained ordination plots), Venn diagrams, heat maps and figures showing the frequency of abundant OTUs. Plots were generated with the ggplot2 package [88].

### Phylogenetic analysis

Phylogenetic analysis of the ITS sequences and of the V3V4 and *rpoB* amplicons, sequence alignment and maximum likelihood analysis with the GTR model were performed as previously described [47]. The Mega7 tool [89] was used to generate a circular phylogenetic tree. Phylogenetic analysis of the 16S rRNA genes from 47 bacterial isolates from *S*. *carpocapsae* IJs, sequence alignment and neighbour-joining analysis with the Kimura two-parameter model were performed as described by Tailliez et al. [90].

## Supplementary information

**Additional file 1.** List of entomopathogenic nematodes used in this study

**Additional file 2. **Rarefaction curves obtained by Illumina-amplicon sequencing of the 16S (**a** and **c**) and *rpoB* (**b** and **d**) markers in various community samples. Rarefaction curves were assembled, with an estimation of species richness (*x*-axis), defined with a sequence identity cutoff of 97%, relative to the total number of bacterial sequences identified (*y*-axis). Samples are presented separately. Sample identities are indicated by specific colours (see legend below the figures).

**Additional file 3. **Box-plots illustrating alpha diversity (observed OTUs and Shannon diversity index) in the microbiota of *Steinernema* IJ samples, *Galleria* samples and control samples obtained by Illumina-amplicon sequencing of the V3V4 region of the 16S rRNA gene (Panels A and B) and the *rpoB* region (Panel C). Median values and interquartile ranges are indicated on the plots. Sample identities are indicated by specific colours (see legend below the figures), three to six technical replicates per sample type were performed. A. V3V4 amplicon sequencing. Estimated OTU richness and diversity indices of *S*. *carpocapsae* SK27 (Batch_23_08_16), *Galleria mellonella* larvae used for IJ multiplication and experimental control samples (Kitome_QE, Kitome_MN, Tap water and Ringer); B. V3V4 amplicon sequencing. Estimated OTU richness and diversity indices of *S*. *carpocapsae* (SK27_23_08_16 and B10_27_04_16); *S*. *weiseri* (583_09_06_15, TCH02_11_08_16, TUR03_21_01_16 and TUR03_09_06_15); *S*. *glaseri* (SK39_09_06_15); *S*. *feltiae* (FRA200_09_06_15 and FRA200_12_08_15) and *H*. *bacteriophora* (TT01_22_06_16 and TT01_15_03_16). C. *rpoB* amplicon sequencing. Estimated OTU richness and diversity indices of *S*. *carpocapsae* (SK27_23_08_16 and B10_27_04_16); *S*. *weiseri* (TCH02_11_08_16); *S*. *glaseri* (SK39_09_06_15); *S*. *feltiae* (FRA200_09_06_15 and FRA200_12_08_15) and *H*. *bacteriophora* (TT01_22_06_16 and TT01_15_03_16).

**Additional file 4. **Diversity and composition of bacterial communities associated with IJs of *S*. *carpocapsae* SK27, to *Galleria mellonella* larvae used for IJ multiplication and to control samples (Kitome_QE, Kitome_MN, Tap water and Ringer). IJ microbiota were studied by metabarcoding with the V3V4 region of the 16S rRNA gene. A. Principal co-ordinates analysis (PCoA) based on Bray-Curtis distances of *S*. *carpocapsae* SK27 batch SK27_23_08_16 samples (squares), *G*. *mellonella* samples (triangles) and control samples (circles). Each point represents an individual replicate. The proportion of the variance explained by each axis is indicated as a percentage. Segregation between the three sample sets is statistically significant (Permanova, Df=2, R^2^=0.51, *p*-value=10^-4^). B and C. Bacterial composition of *S*. *carpocapsae* SK27 batch SK27_23_08_16 samples, *G*. *mellonella* samples and control samples. Bar plots, each representing an individual replicate, showing the relative abundance of (B) the five most frequently represented OTUs at the phylum level (Top 5 phylum), (C) the nine most frequently represented OTUs at the family level (Top 9 family). D. Heatmap showing the microbiota composition of *Galleria* samples (whole insects). Each column represents a *Galleria* sample. The 30 most abundant OTUs across the samples at the genus affiliation level (Top30 Genus) are listed on the left. The percentage relative abundance is indicated by the gradient of blue hues.

**Additional file 5. **Comparison of the microbiota of *S*. *carpocapase* SK27 batch SK27_23_08_16 (six technical replicates) based on two taxonomic markers, the V3V4 region of the 16S rRNA gene (A) and the 430bp-*rpoB* region (B). Phylogenetic trees of OTUs based on the *rpoB* 435 bp region or the V3V4 region of the 16S rRNA gene were inferred with SEAVIEW 4.0 [91], using a PhyML-based maximum likelihood algorithm [92] and the GTR model. The sum of read numbers for the six replicates is indicated after the OTU name. Only abundant OTUs with read numbers accounting for more than 0. 1% of the reads for the sample in at least one replicate are included in the phylogenetic tree. OTUs belonging to the same bacterial genus are shown in the same colour.

**Additional file 6. **Comparison of bacterial communities associated with IJs from different genera (*Steinernema* and *Heterorhabditis*) and different species (*S*. *carpocapsae*, *S*. *feltiae*, *S*. *weiseri* and *S*. *glaseri*). A. Principal coordinates analysis (PCoA) based on Bray-Curtis distances for IJ microbiota based on the V3V4 region of the 16S rRNA gene. *Heterorhabditis* and *Steinernema* samples are indicated with circles and triangles, respectively. Colors indicate the different *Steinernema* species. Each point represents a technical replicate. The proportion of the variance explained by each axis is shown. The five samples are statistically different (Permanova *rpoB*, Df=4, R^2^=0.73, *p*-value=10^-4^). B. Heatmap showing the microbiota composition of IJ samples based on the V3V4 region of the 16S rRNA gene. Each column represents an IJ species. The 30 most abundant OTUs across the samples at the genus affiliation level (Top30 Genus) are listed on the left. The percentage relative abundance is indicated by the gradient of blue hues. C. Heatmap showing the microbiota composition of different strains of *Steinernema carpocapsae* based on the V3V4 region of the 16S rRNA gene and the 435 bp *rpoB* region. Each column represents an IJ species. The 30 most abundant OTUs across the samples at the species affiliation level (Top30 species) are listed on the left. The percentage relative abundance is indicated by the gradient of blue hues. The nematode strains used here belong to the following batches: *S*. *carpocapsae* SK27_23_08_16 and B10_27_04_16; *S*. *weiseri* 583_09_06_15, TCH02_11_08_16 (t1), TUR03_21_01_16 and TUR03_09_06_15; *S*. *glaseri* SK39_09_06_15; *S*. *feltiae* FRA200_09_06_15 and FRA200_12_08_15 and *H*. *bacteriophora* TT01_22_06_16 and TT01_15_03_16. Three to six technical replicates per batch were performed.

**Additional file 7. **Principal coordinates analysis (PCoA) based on Bray-Curtis distances for IJ microbiota obtained by metabarcoding with the V3V4 region of the 16S rRNA gene. Each point represents an individual sample replicate. The different strains, multiplication batches and origins of *S*. *carpocapsae* are indicated by colours and symbols. The proportion of the variance explained by each axis is shown. A. Comparison of six *S*. *carpocapsae* strains. The six samples are significantly different (Permanova, Df=5, R^2^=0.75, *p*-value=10^-4^). B. Comparison of four multiplication batches of *S*. *carpocapsae* SK27. The four samples are significantly different (Permanova, Df=3, R^2^=0.73, *p*-value=10^-4^). C. Comparison of two laboratory origins of *S*. *carpocapsae* DD136 and *S*. *carpocapsae* All (Permanova: DD136 versus All, Df=1, R^2^=0.09, *p*-value=10^-4^; DGIMI versus USDA, Df=1, R^2^=0.28, *p*-value=10^-4^).

**Additional file 8. **Heatmap showing the microbiota composition of *Steinernema carpocapsae* strains. Each column represents a replicate (strain or batch). The 30 most abundant OTUs across the samples at the species affiliation level (**a** and **c**) and at the genus affiliation level (**b** and **d**) for the the 435 bp *rpoB* region (A and B) and the V3V4 region of the 16S gene (**c** and **d**) are listed on the left. The percentage of relative abundance is indicated by the gradient of blue hues.

**Additional file 9. **Heatmap showing the microbiota composition of the *S*. *carpocapsae* GRAB strain freshly isolated (November 2017) from the soil of an apple orchard in Gard, France. Each column represents a technical replicate. The 30 most abundant OTUs across the samples at the species affiliation level for the 435 bp *rpoB* region or the V3V4 region of the 16S gene are listed on the left. The percentage of relative abundance is indicated by the gradient of blue hues.

**Additional file 10. **Antimicrobial activities of *Xenorhabdus* (**a**) and *Pseudomonas* (**b**) strains against bacterial taxa from the IJ-associated microbiota of *S*. *carpocapsae* SK27, *S*. *glaseri* SK39 and *S*. *weiseri* 583.

**Additional file 11.** Bacterial strains isolated in the study and used in pathology and/or antibiosis assays.

**Additional file 12. **List of the 62 bacterial strains, including 50 bacterial isolates from *S*.*carpocapsae* IJs, used in this study to build the 16S rRNA gene phylogenetic tree.

**Additional file 13. a**. List of OTUs obtained with the V3V4 marker. **b**. List of OTUs obtained with *rpoB* marker. **c**. Venn diagram table detailing compositions of core and non-core OTUs of Figure 2a. **d**. List and frequency of OTUs associated with the *S*. *carpocapsae* IJs obtained with the V3V4 marker for which abundance in the whole dataset exceeded 0.1% (plotted in Figure 5.b). **e**. List and frequency of OTUs associated with *S*. *carpocapsae* IJs obtained with the *rpoB* marker for which abundance in the whole dataset exceeded 0.1% (plotted in Figure 5.a).

## Data Availability

Raw data of the MiSeq sequencing are available at the European Archive database (BioProject PRJEB24936, http://www.ebi.ac.uk/ena/data/view/PRJEB24936). Accession numbers of the 16S RNA genes used for phylogenetic tree in Fig. 6 are listed in Additional file 12. The abundance tables of OTUs with the V3V4 and the *rpoB* markers as well as the lists of OTUs used for Figs. 2a and 5 are available in Additional file 13.
