# Supplementary material for: Entomopathogenic nematode-associated microbiota: from monoxenic paradigm to pathobiome
Source: Microbiome. 2020 Feb 24;8:25. doi: 10.1186/s40168-020-00800-5 (PMC7041241; doi:10.1186/s40168-020-00800-5)
Supplement: Supplementary file 1 — Additional file 1. List of entomopathogenic nematodes used in this study [file 40168_2020_800_MOESM1_ESM.pdf]

Additional File 1: List of entomopathogenic nematodes used in this study

Nematode	Geographic origin	Laboratory history	Multiplication batch (dd_mm_yy)
Steinernema carpocapsae			
SK27	Plougastel, France	>20 years in lab collection (Montpellier, France)	09_06_15<br>15_03_16<br>23_08_16
		SK27 strain transferred to laboratory of J.B Ferdy for two years (Toulouse, France)	27_07_16
SK27_ApoII	Plougastel, France	Offspring of SK27 eggs disinfected with 0.24 % sodium hypochlorite; stored for 3 years in laboratory collection (Montpellier, France)	07_11_16
EGY03 (Egypt2)	Egypt	5 years in the collection of V. Puza (Hungary) and stored for 2 years in laboratory collection (Montpellier, France)	20_07_16 (t1)
CREA3	Haute-Savoie, France	4 years in CREA collection (Bonneville, France)	13_07_16 (t0)<br>27_07_16 (t1)
B10 (FRA36)	Avignon, France	10 years in laboratory collection (Montpellier, France)	27_04_16
All_DGIMI	Georgia, USA	9 years in laboratory collection (Montpellier, France)	01_06_16
All_USDA	Georgia, USA	>20 years in the collection of D. Shapiro (Byron, USA)	21_10_16 (t0)<br>07_11_16 (t1)
DD136_DGIMI	Georgia, USA	9 years in laboratory collection (Montpellier, France)	24_04_16
DD136_USDA	Georgia, USA	>20 years in the collection of D. Shapiro (Byron, USA)	21_10_16 (t0)<br>07_11_16 (t1)
GRAB (FRA241)	Avignon, France	Isolated in November 2017 (Montpellier, France)	22_12_17
Steinernema weiseri			
583	Czech Republic	12 years in laboratory collection (Montpellier, France)	20_07_16<br>09_06_15
TCH02	Czech Republic	2 years in laboratory collection (Montpellier, France)	11_08_16 (t1)
TUR03	Turkey	7 years in laboratory collection (Montpellier, France)	09_06_15<br>21_01_16

Steinernema glaseri			
SK39	North Carolina, USA	10 years in lab collection (Montpellier, France)	18_08_16<br>09_06_15
Steinernema feltiae			
FRA200	Hérault, France	5 years in lab collection (Montpellier, France)	09_06_15<br>12_08_15
Heterorhabditis bacteriophora			
TT01	Trinidad & Tobago	10 years in lab collection (Montpellier, France)	09_06_15<br>15_03_16<br>22_06_16
