# Supplementary figures and images for: Entomopathogenic nematode-associated microbiota: from monoxenic paradigm to pathobiome
Source: Microbiome. 2020 Feb 24;8:25. doi: 10.1186/s40168-020-00800-5 (PMC7041241; doi:10.1186/s40168-020-00800-5)

## Additional File 2

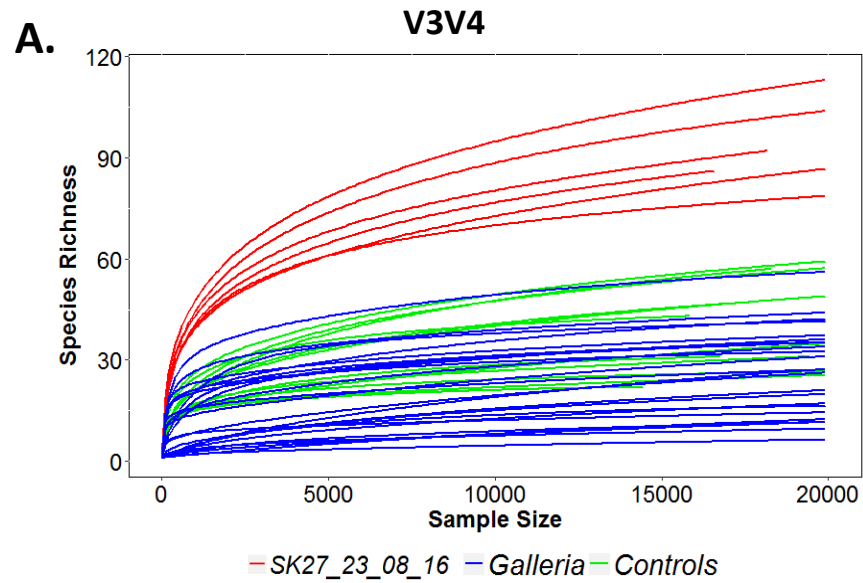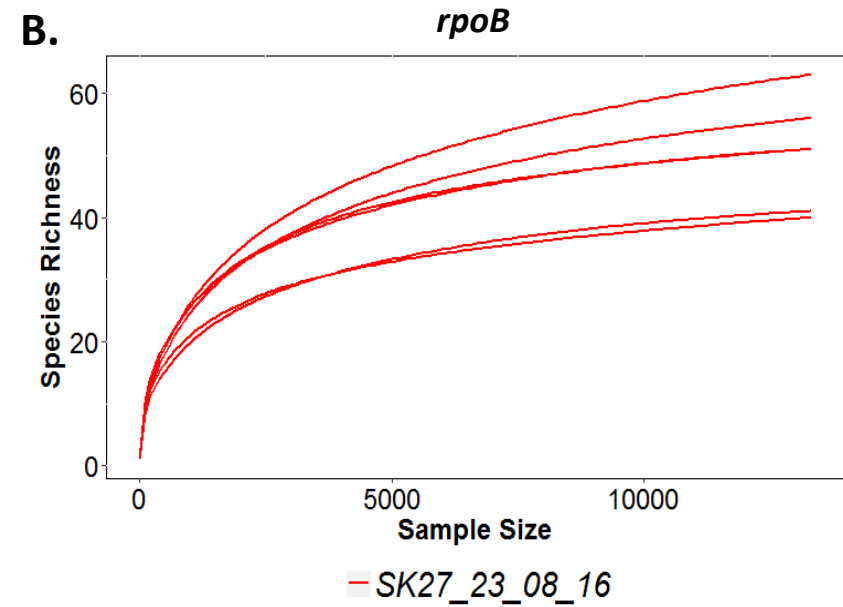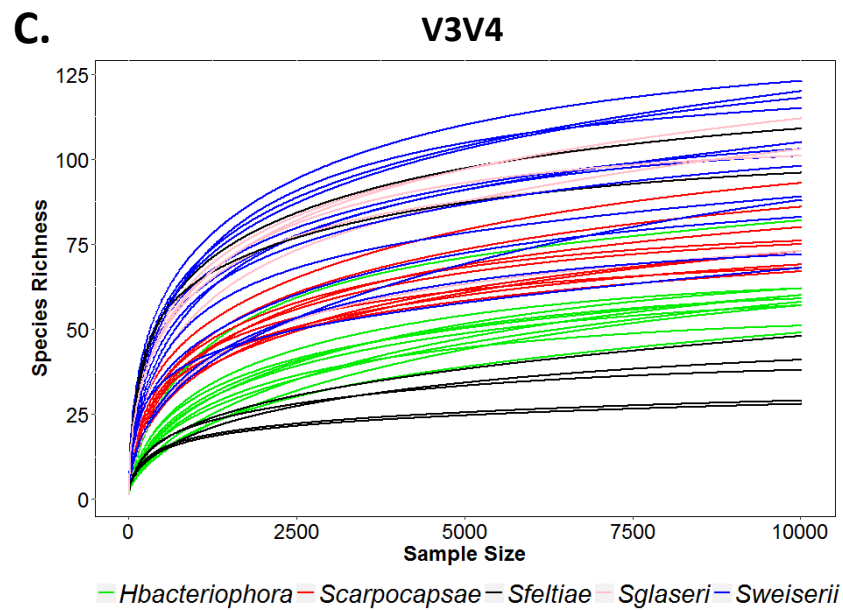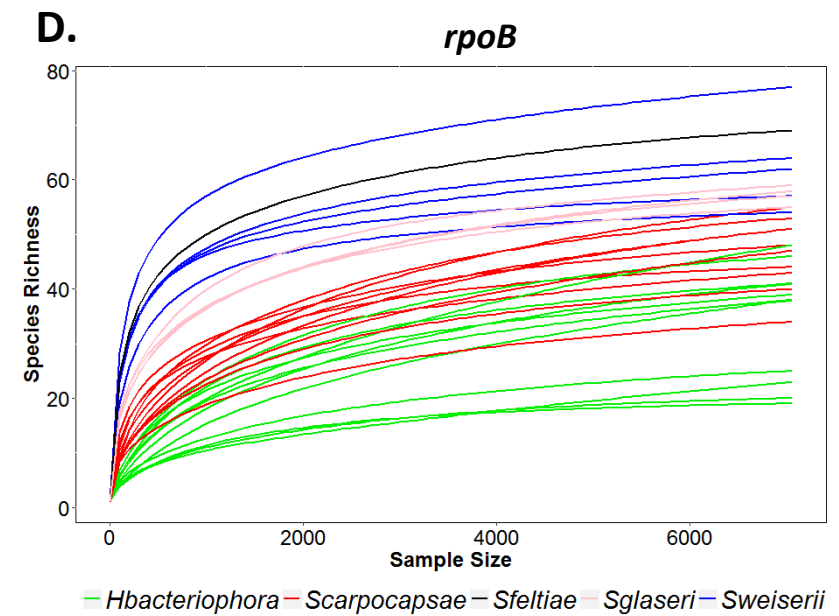

Supplement: Supplementary file 2 — Additional file 2. Rarefaction curves obtained by Illumina-amplicon sequencing of the 16S (a and c) and rpoB (b and d) markers in various community samples. Rarefaction curves were assembled, with an estimation of species richness (x-axis), defined with a sequence identity cutoff of 97%, relative to the total number of bacterial sequences identified (y-axis). Samples are presented separately. Sample identities are indicated by specific colours (see legend below the figures). [file 40168_2020_800_MOESM2_ESM.pdf]

Additional File 4

A.

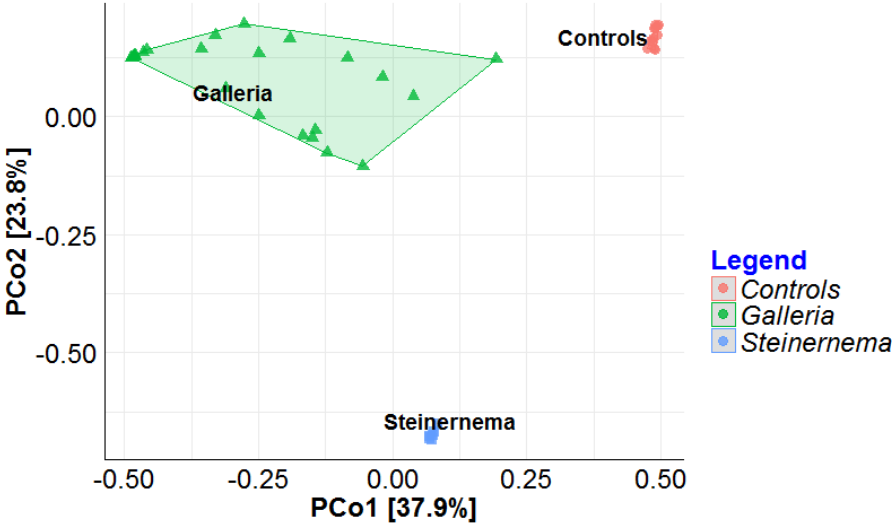

B.

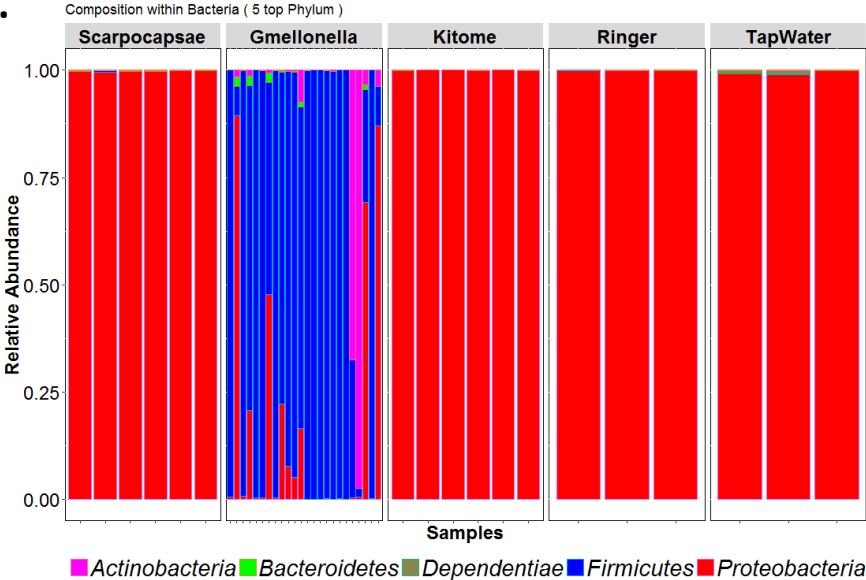

C.

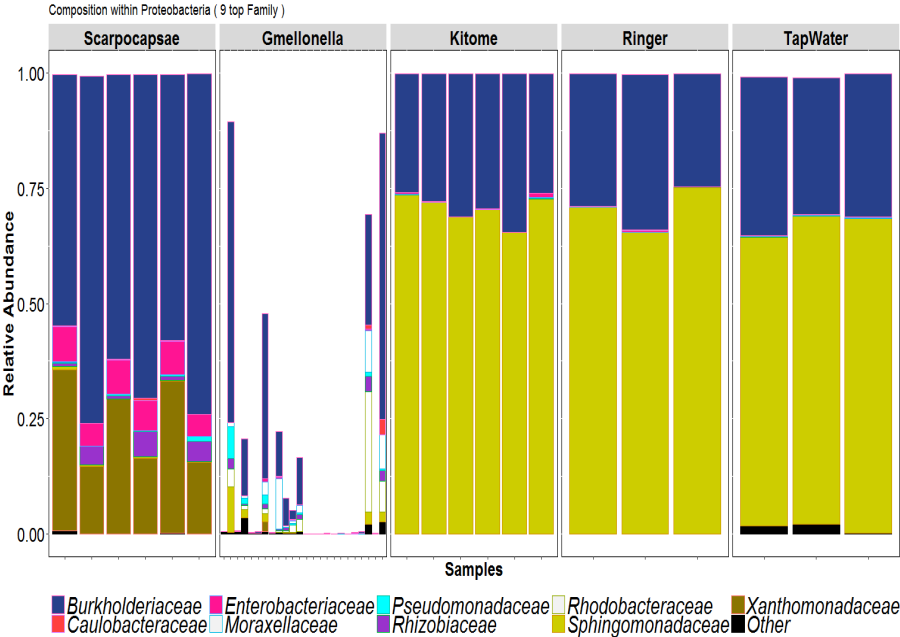

D.

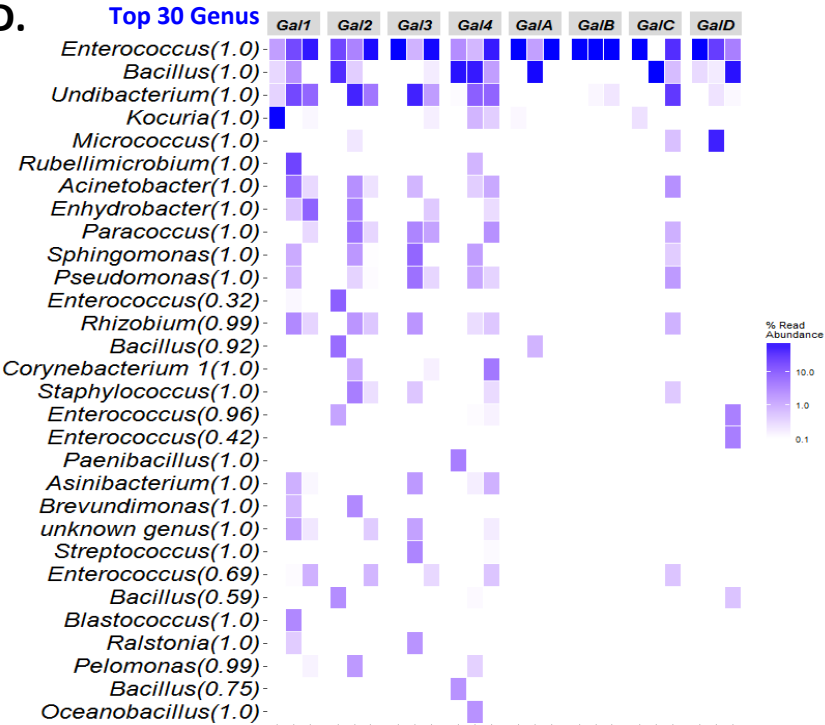

Supplement: Supplementary file 4 — Additional file 4. Diversity and composition of bacterial communities associated with IJs of S. carpocapsae SK27, to Galleria mellonella larvae used for IJ multiplication and to control samples (Kitome_QE, Kitome_MN, Tap water and Ringer). IJ microbiota were studied by metabarcoding with the V3V4 region of the 16S rRNA gene. A. Principal co-ordinates analysis (PCoA) based on Bray-Curtis distances of S. carpocapsae SK27 batch SK27_23_08_16 samples (squares), G. mellonella samples (triangles) and control samples (circles). Each point represents an individual replicate. The proportion of the variance explained by each axis is indicated as a percentage. Segregation between the three sample sets is statistically significant (Permanova, Df=2, R2=0.51, p-value=10-4). B and C. Bacterial composition of S. carpocapsae SK27 batch SK27_23_08_16 samples, G. mellonella samples and control samples. Bar plots, each representing an individual replicate, showing the relative abundance of (B) the five most frequently represented OTUs at the phylum level (Top 5 phylum), (C) the nine most frequently represented OTUs at the family level (Top 9 family). D. Heatmap showing the microbiota composition of Galleria samples (whole insects). Each column represents a Galleria sample. The 30 most abundant OTUs across the samples at the genus affiliation level (Top30 Genus) are listed on the left. The percentage relative abundance is indicated by the gradient of blue hues. [file 40168_2020_800_MOESM4_ESM.pdf]

## Additional File 5

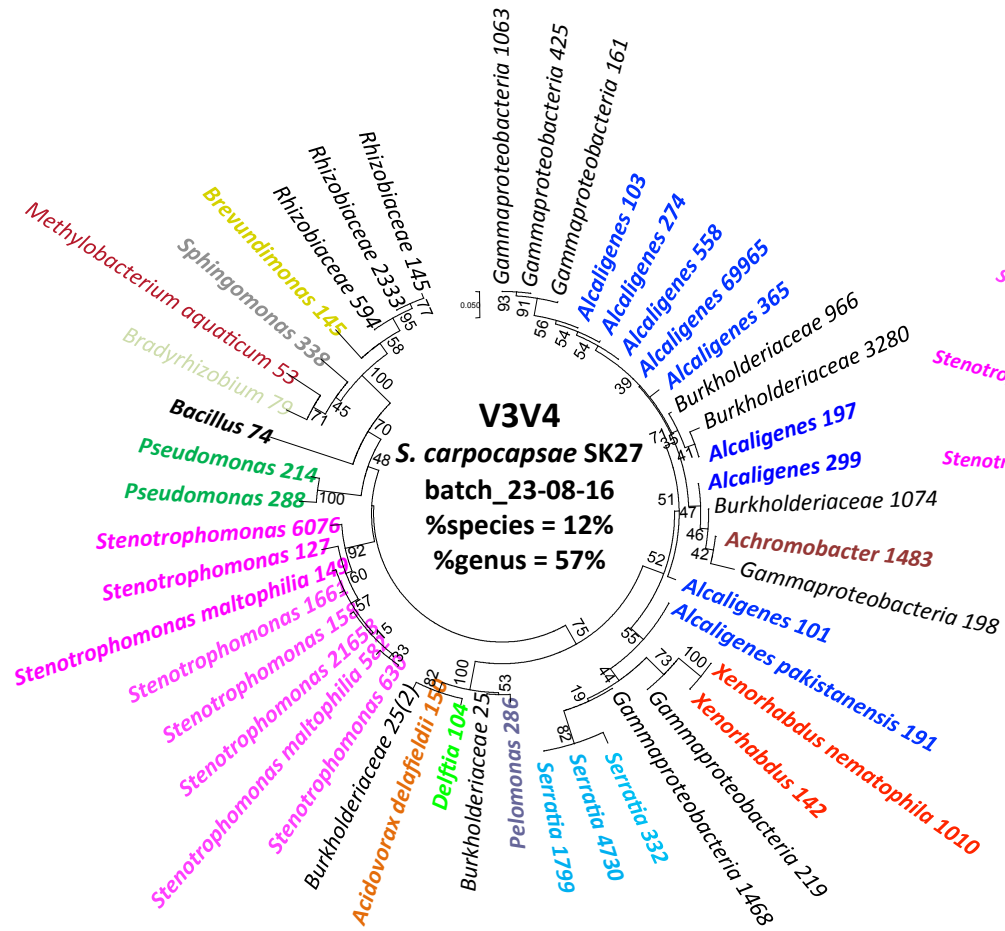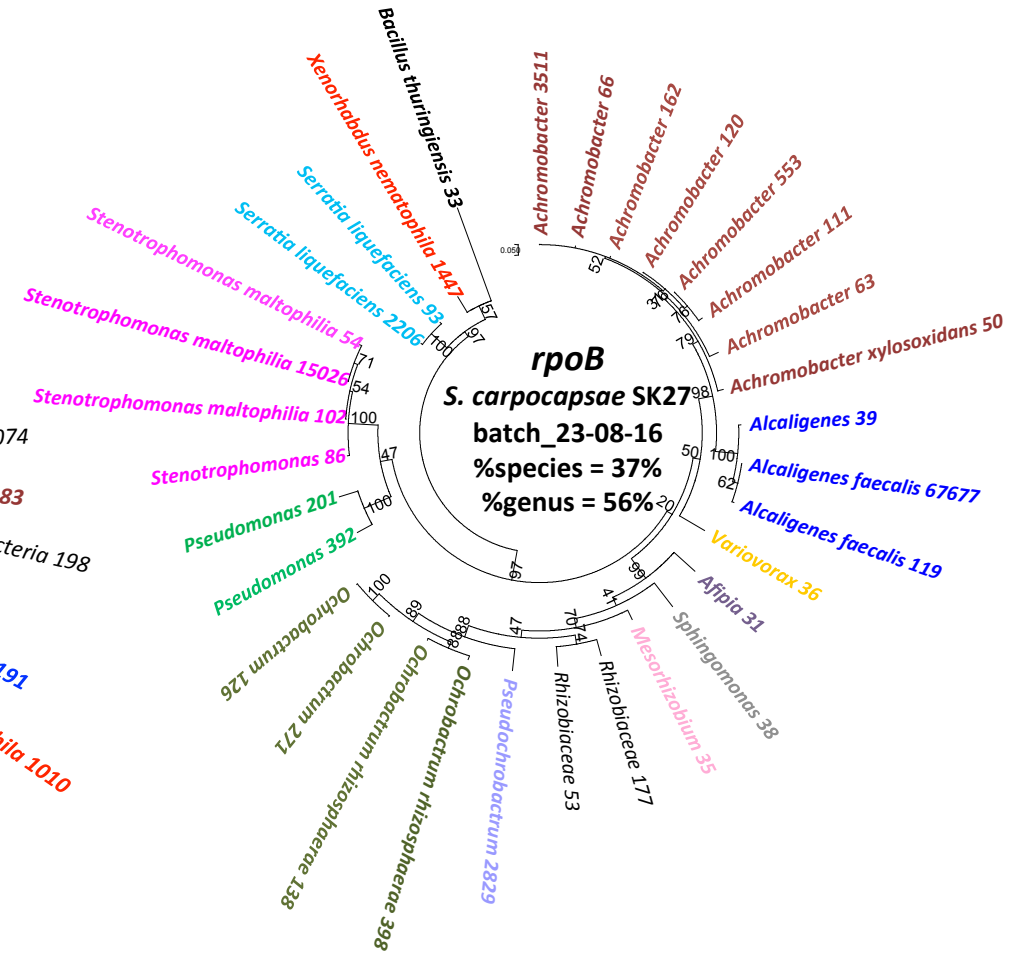

Supplement: Supplementary file 5 — Additional file 5. Comparison of the microbiota of S. carpocapase SK27 batch SK27_23_08_16 (six technical replicates) based on two taxonomic markers, the V3V4 region of the 16S rRNA gene (A) and the 430bp-rpoB region (B). Phylogenetic trees of OTUs based on the rpoB 435 bp region or the V3V4 region of the 16S rRNA gene were inferred with SEAVIEW 4.0 [91], using a PhyML-based maximum likelihood algorithm [92] and the GTR model. The sum of read numbers for the six replicates is indicated after the OTU name. Only abundant OTUs with read numbers accounting for more than 0. 1% of the reads for the sample in at least one replicate are included in the phylogenetic tree. OTUs belonging to the same bacterial genus are shown in the same colour. [file 40168_2020_800_MOESM5_ESM.pdf]

Additional File 6

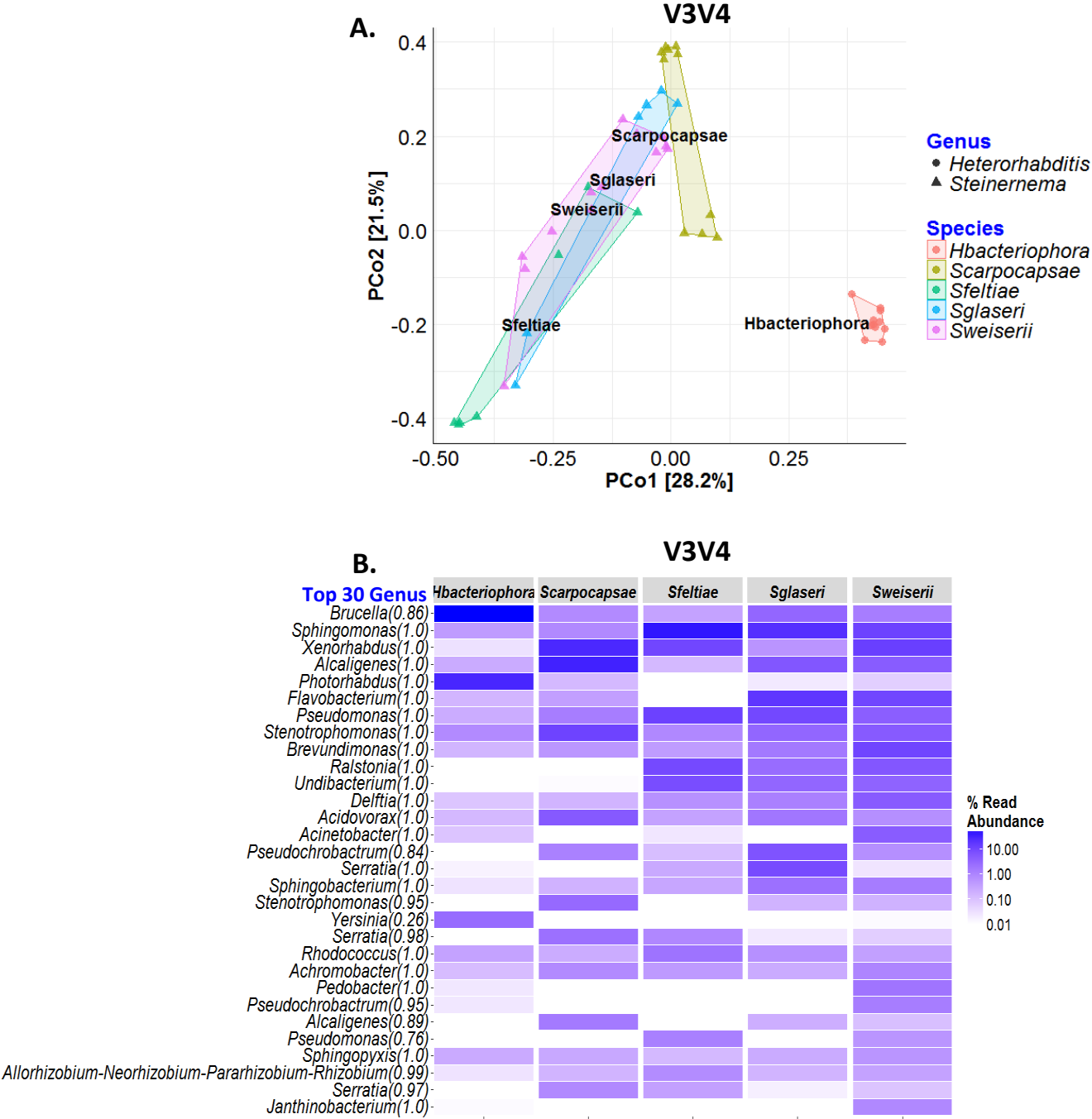

C.

## Top 30 species

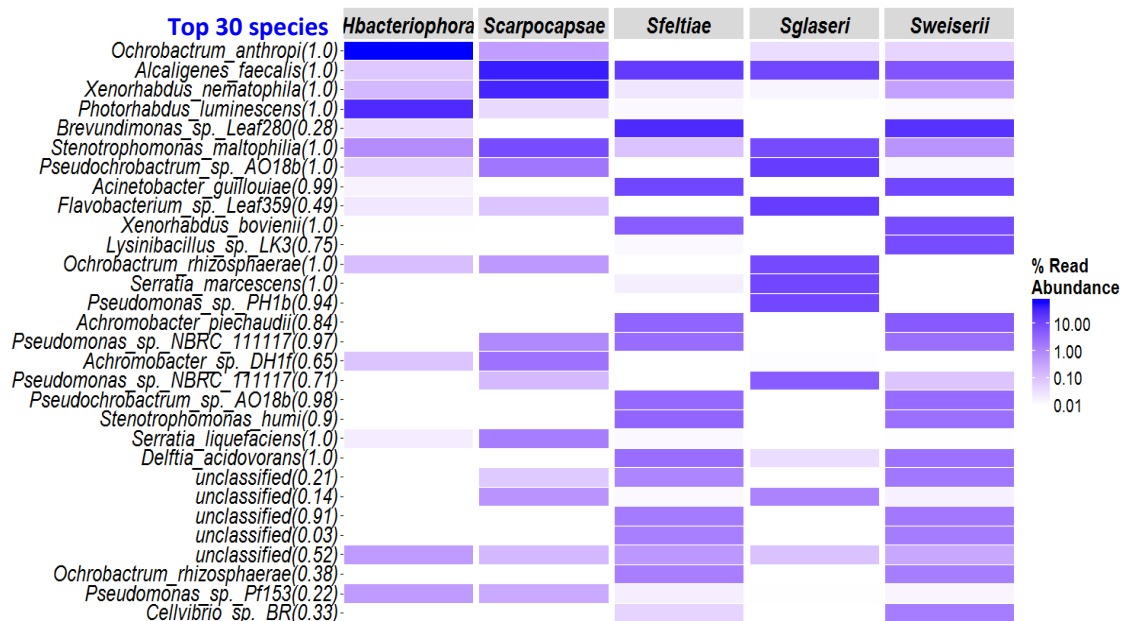

% Read  
Abundance

10.00  
1.00  
0.10  
0.01

rpoB

## Top 30 species

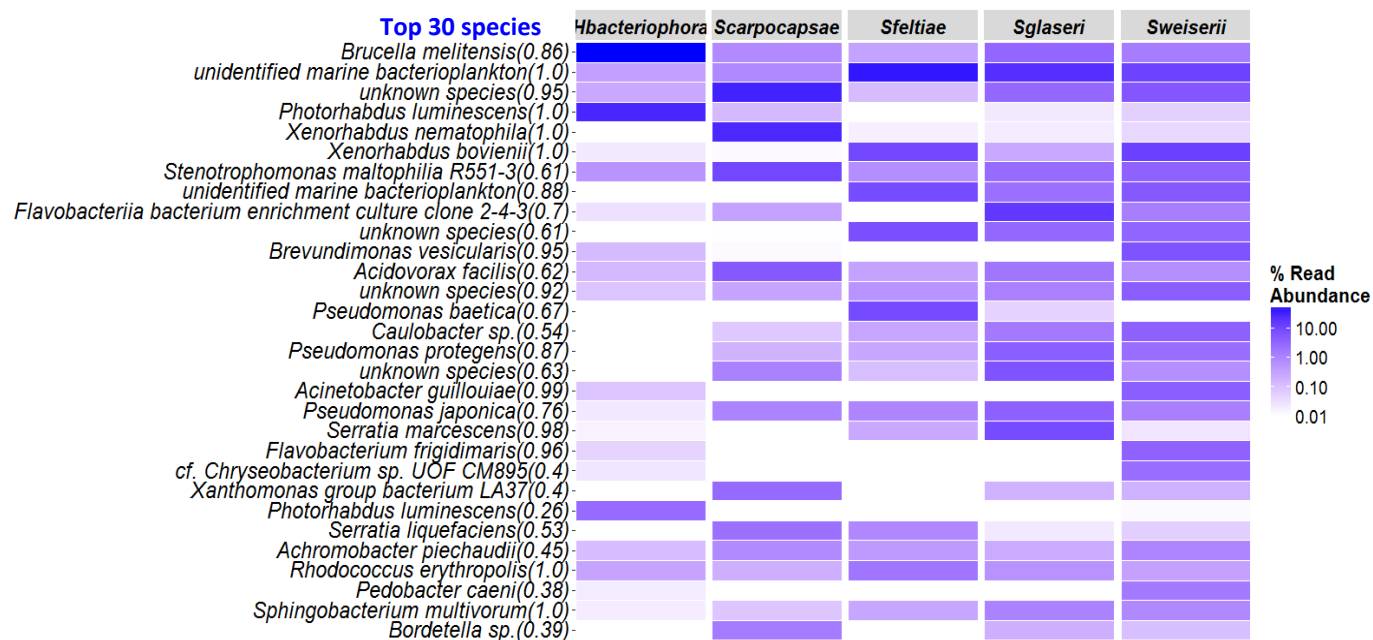

% Read  
Abundance

10.00  
1.00  
0.10  
0.01

V3V4

Supplement: Supplementary file 6 — Additional file 6. Comparison of bacterial communities associated with IJs from different genera (Steinernema and Heterorhabditis) and different species (S. carpocapsae, S. feltiae, S. weiseri and S. glaseri). A. Principal coordinates analysis (PCoA) based on Bray-Curtis distances for IJ microbiota based on the V3V4 region of the 16S rRNA gene. Heterorhabditis and Steinernema samples are indicated with circles and triangles, respectively. Colors indicate the different Steinernema species. Each point represents a technical replicate. The proportion of the variance explained by each axis is shown. The five samples are statistically different (Permanova rpoB, Df=4, R2=0.73, p-value=10-4). B. Heatmap showing the microbiota composition of IJ samples based on the V3V4 region of the 16S rRNA gene. Each column represents an IJ species. The 30 most abundant OTUs across the samples at the genus affiliation level (Top30 Genus) are listed on the left. The percentage relative abundance is indicated by the gradient of blue hues. C. Heatmap showing the microbiota composition of different strains of Steinernema carpocapsae based on the V3V4 region of the 16S rRNA gene and the 435 bp rpoB region. Each column represents an IJ species. The 30 most abundant OTUs across the samples at the species affiliation level (Top30 species) are listed on the left. The percentage relative abundance is indicated by the gradient of blue hues. The nematode strains used here belong to the following batches: S. carpocapsae SK27_23_08_16 and B10_27_04_16; S. weiseri 583_09_06_15, TCH02_11_08_16 (t1), TUR03_21_01_16 and TUR03_09_06_15; S. glaseri SK39_09_06_15; S. feltiae FRA200_09_06_15 and FRA200_12_08_15 and H. bacteriophora TT01_22_06_16 and TT01_15_03_16. Three to six technical replicates per batch were performed. [file 40168_2020_800_MOESM6_ESM.pdf]

## Additional File 7

**A.**

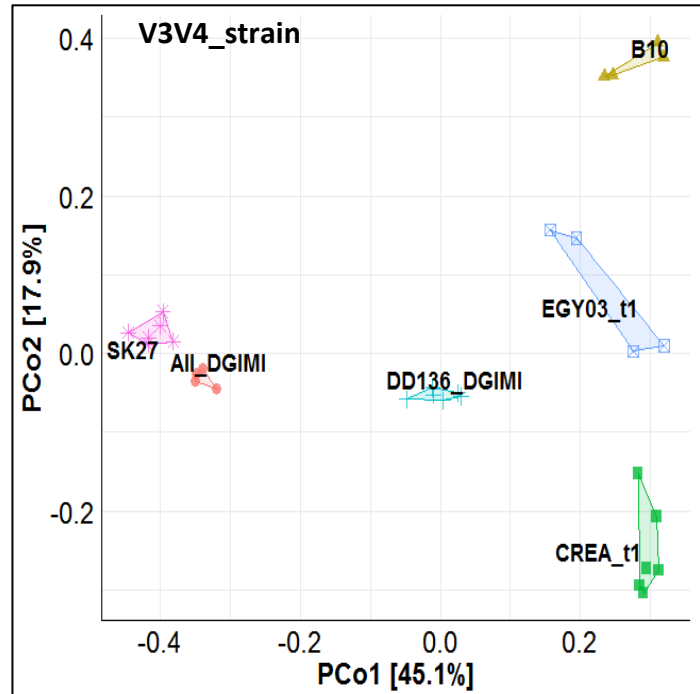

**B.**

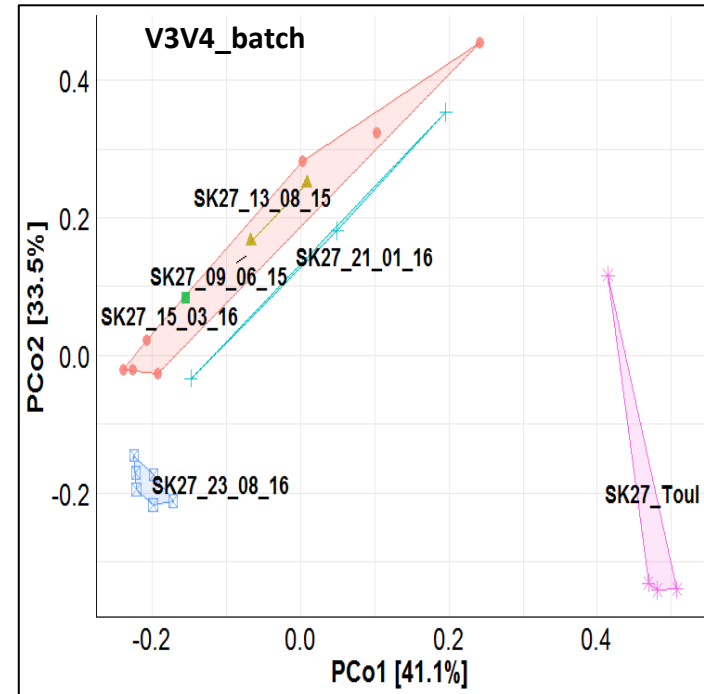

**C.**

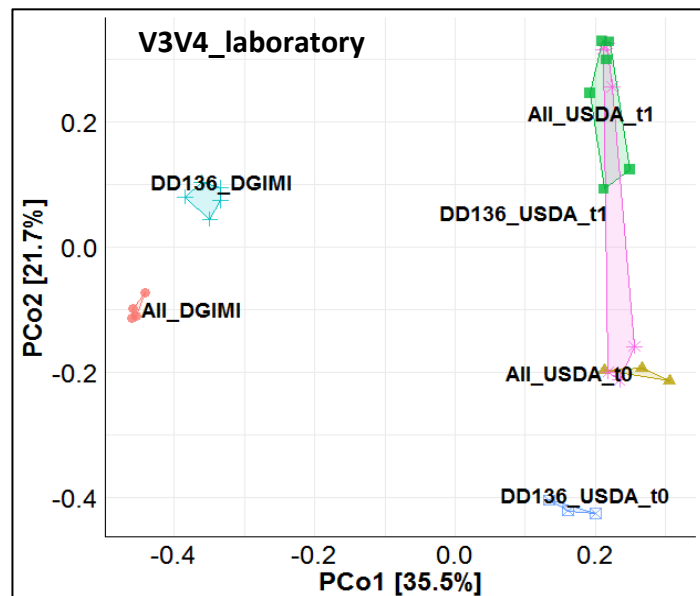

Supplement: Supplementary file 7 — Additional file 7. Principal coordinates analysis (PCoA) based on Bray-Curtis distances for IJ microbiota obtained by metabarcoding with the V3V4 region of the 16S rRNA gene. Each point represents an individual sample replicate. The different strains, multiplication batches and origins of S. carpocapsae are indicated by colours and symbols. The proportion of the variance explained by each axis is shown. A. Comparison of six S. carpocapsae strains. The six samples are significantly different (Permanova, Df=5, R2=0.75, p-value=10-4). B. Comparison of four multiplication batches of S. carpocapsae SK27. The four samples are significantly different (Permanova, Df=3, R2=0.73, p-value=10-4). C. Comparison of two laboratory origins of S. carpocapsae DD136 and S. carpocapsae All (Permanova: DD136 versus All, Df=1, R2=0.09, p-value=10-4; DGIMI versus USDA, Df=1, R2=0.28, p-value=10-4). [file 40168_2020_800_MOESM7_ESM.pdf]

Additional File 9

*rpoB*

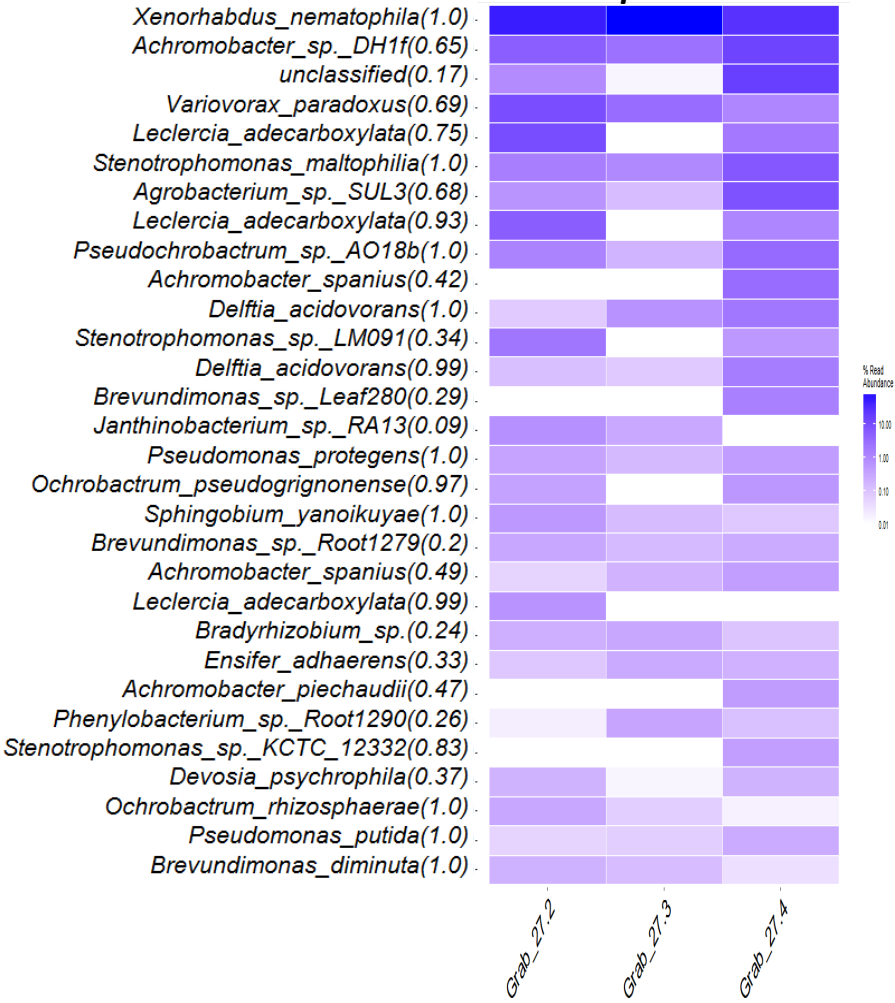

V3V4

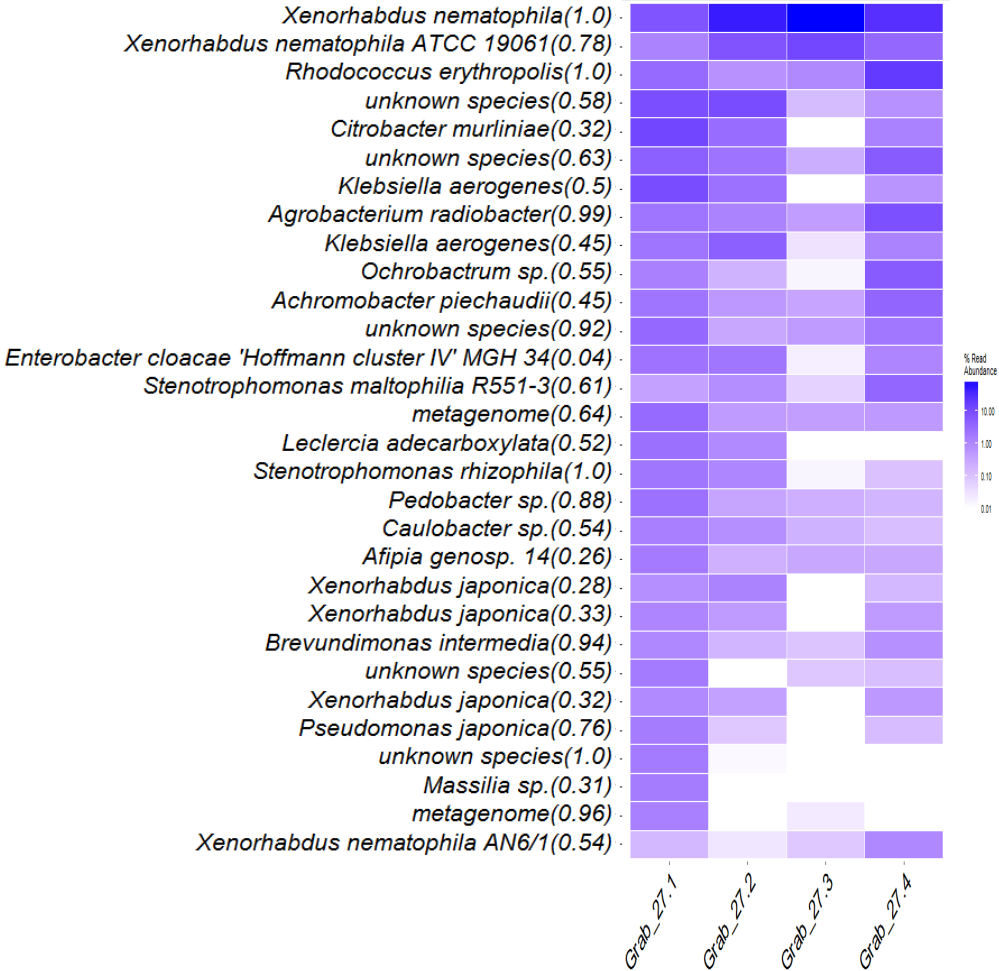

Supplement: Supplementary file 9 — Additional file 9. Heatmap showing the microbiota composition of the S. carpocapsae GRAB strain freshly isolated (November 2017) from the soil of an apple orchard in Gard, France. Each column represents a technical replicate. The 30 most abundant OTUs across the samples at the species affiliation level for the 435 bp rpoB region or the V3V4 region of the 16S gene are listed on the left. The percentage of relative abundance is indicated by the gradient of blue hues. [file 40168_2020_800_MOESM9_ESM.pdf]
