# Supplementary material for: Entomopathogenic nematode-associated microbiota: from monoxenic paradigm to pathobiome
Source: Microbiome. 2020 Feb 24;8:25. doi: 10.1186/s40168-020-00800-5 (PMC7041241; doi:10.1186/s40168-020-00800-5)

Additional File 3

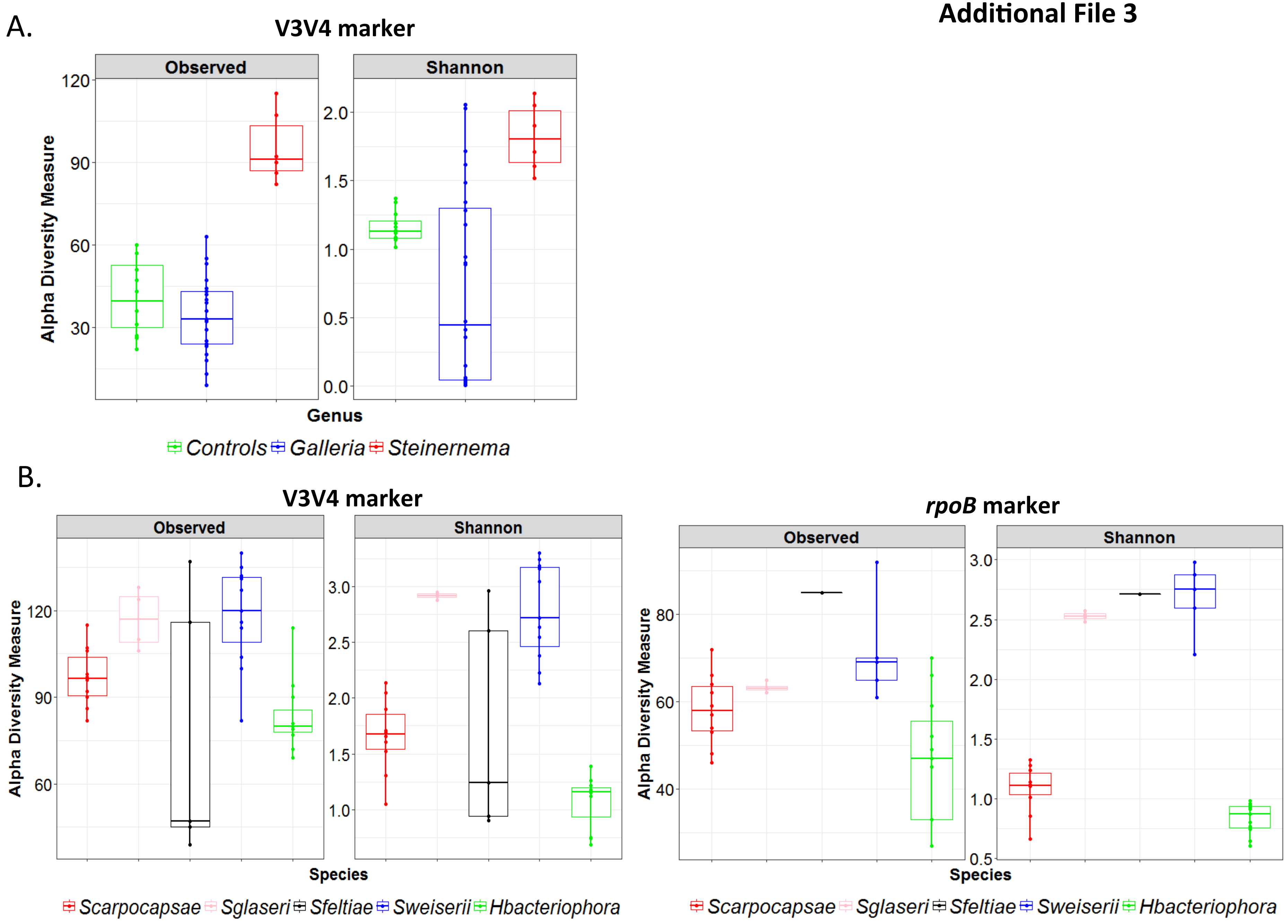

Supplement: Supplementary file 3 — Additional file 3. Box-plots illustrating alpha diversity (observed OTUs and Shannon diversity index) in the microbiota of Steinernema IJ samples, Galleria samples and control samples obtained by Illumina-amplicon sequencing of the V3V4 region of the 16S rRNA gene (Panels A and B) and the rpoB region (Panel C). Median values and interquartile ranges are indicated on the plots. Sample identities are indicated by specific colours (see legend below the figures), three to six technical replicates per sample type were performed. A. V3V4 amplicon sequencing. Estimated OTU richness and diversity indices of S. carpocapsae SK27 (Batch_23_08_16), Galleria mellonella larvae used for IJ multiplication and experimental control samples (Kitome_QE, Kitome_MN, Tap water and Ringer); B. V3V4 amplicon sequencing. Estimated OTU richness and diversity indices of S. carpocapsae (SK27_23_08_16 and B10_27_04_16); S. weiseri (583_09_06_15, TCH02_11_08_16, TUR03_21_01_16 and TUR03_09_06_15); S. glaseri (SK39_09_06_15); S. feltiae (FRA200_09_06_15 and FRA200_12_08_15) and H. bacteriophora (TT01_22_06_16 and TT01_15_03_16). C. rpoB amplicon sequencing. Estimated OTU richness and diversity indices of S. carpocapsae (SK27_23_08_16 and B10_27_04_16); S. weiseri (TCH02_11_08_16); S. glaseri (SK39_09_06_15); S. feltiae (FRA200_09_06_15 and FRA200_12_08_15) and H. bacteriophora (TT01_22_06_16 and TT01_15_03_16). [file 40168_2020_800_MOESM3_ESM.pdf]
