# Supplementary material for: Entomopathogenic nematode-associated microbiota: from monoxenic paradigm to pathobiome
Source: Microbiome. 2020 Feb 24;8:25. doi: 10.1186/s40168-020-00800-5 (PMC7041241; doi:10.1186/s40168-020-00800-5)

Additional File 8

A. Microbiota composition of the *S. carpocapsae* strains (Top 30 genus)

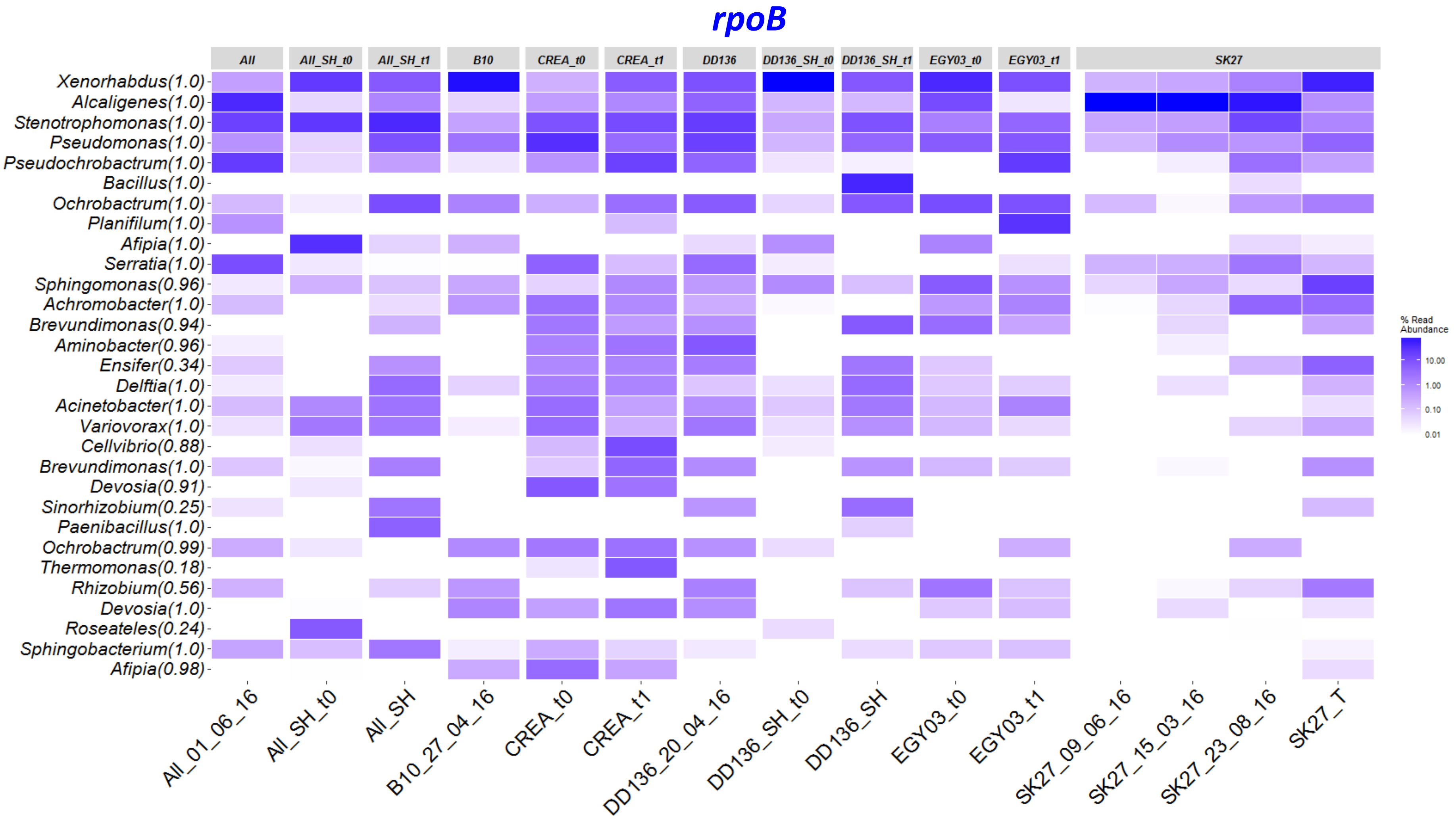

B. Microbiota composition of the *S. carpocapsae* strains (Top 30 species)

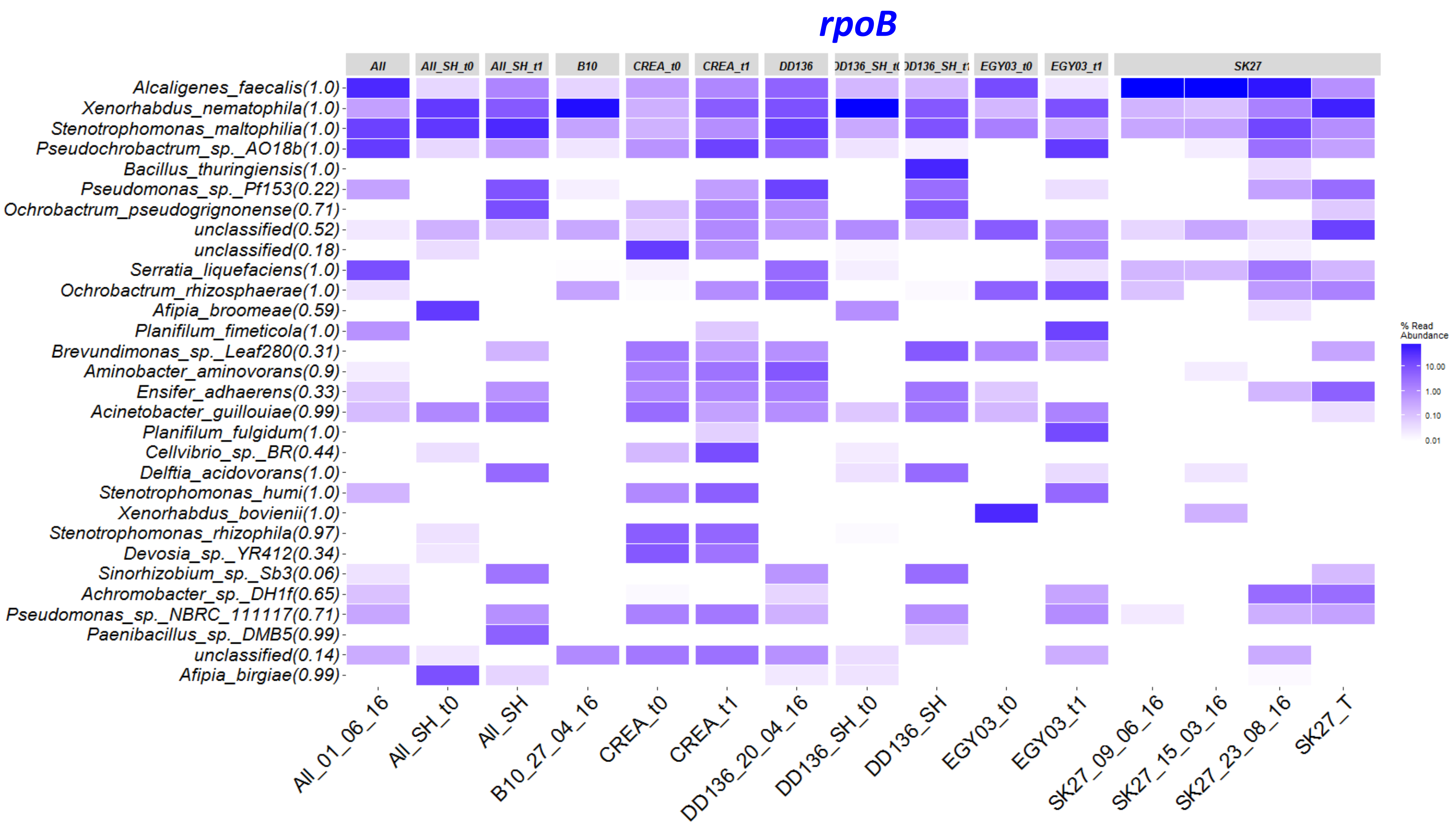

C. Microbiota composition of the *S. carpocapsae* strains (Top 30 genus)

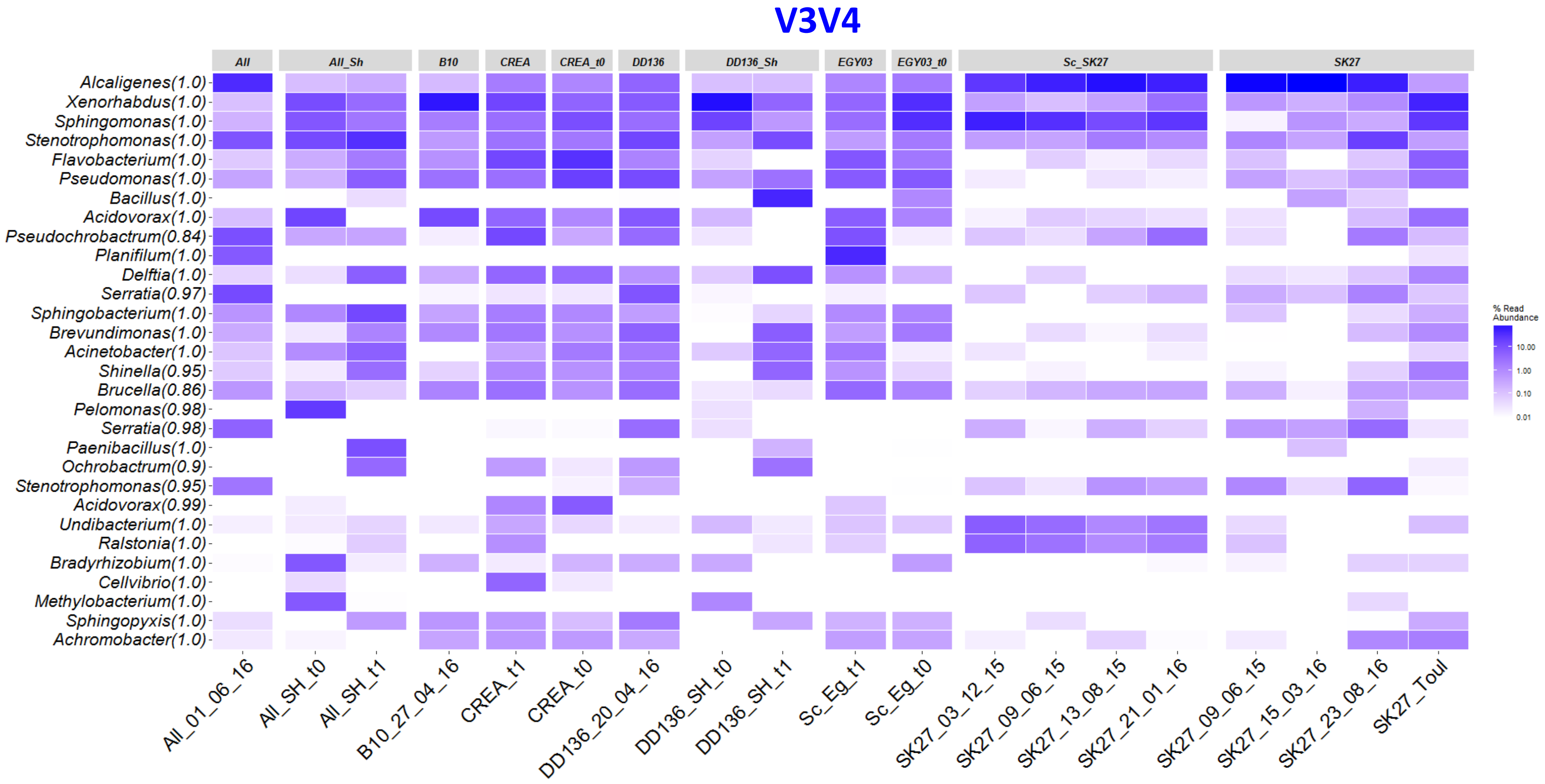

D. Microbiota composition of the *S. carpocapsae* strains (Top 30 species)

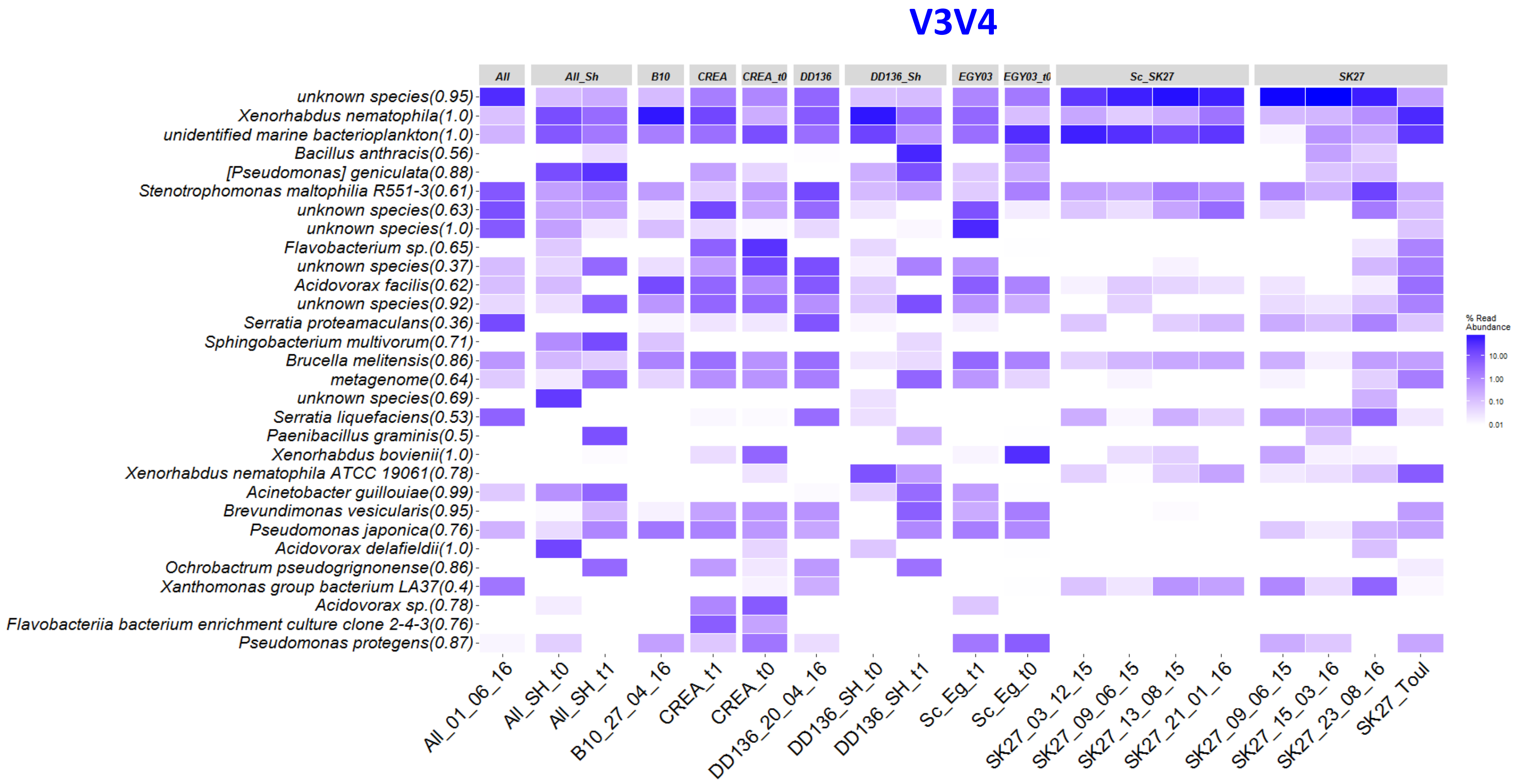

Supplement: Supplementary file 8 — Additional file 8. Heatmap showing the microbiota composition of Steinernema carpocapsae strains. Each column represents a replicate (strain or batch). The 30 most abundant OTUs across the samples at the species affiliation level (a and c) and at the genus affiliation level (b and d) for the the 435 bp rpoB region (A and B) and the V3V4 region of the 16S gene (c and d) are listed on the left. The percentage of relative abundance is indicated by the gradient of blue hues. [file 40168_2020_800_MOESM8_ESM.pdf]
