# Supplementary material for: Entomopathogenic nematode-associated microbiota: from monoxenic paradigm to pathobiome
Source: Microbiome. 2020 Feb 24;8:25. doi: 10.1186/s40168-020-00800-5 (PMC7041241; doi:10.1186/s40168-020-00800-5)
Supplement: Supplementary file 10 — Additional file 10. Antimicrobial activities of Xenorhabdus (a) and Pseudomonas (b) strains against bacterial taxa from the IJ-associated microbiota of S. carpocapsae SK27, S. glaseri SK39 and S. weiseri 583. [file 40168_2020_800_MOESM10_ESM.pdf]

Additional File 10: Antimicrobial activities of *Xenorhabdus* (A) and *Pseudomonas* (B) strains against bacterial taxa from the IJ-associated microbiota of *S. carpocapsae*, *S. glaseri* and *S. weiseri*.

A.

	Positive control	*S carpocapsae* microbiota							*S glaseri* microbiota					*S weiseri* microbiota					
Indicator strains / Producer strains	*Micrococcus luteus* ML	*Xenorhabdus nematophila* XnSc_F1	*Pseudomonas protegens* PpSc_PP-SC-10	*Achromobacter sp* AchSc_D7-1	*Alcaligenes faecalis* AlcfSc_SC	*Ochrobactrum sp* OchSc_ALL4	*Pseudochrobactrum* PochSc_AL3	*Stenotrophomonas maltophilia* StmSc_ALL5	*Xenorhabdus poinarii* XpSg_G6	*Pseudomonas protegens* PpSg_SG6 Apo	*Pseudomonas chlororaphis* PcSg_SK39 ApoA	*Stenotrophomonas maltophilia* StmSg_SK39-2	*Achromobacter sp* AchSg_SK39 ApoC	*Xenorhabdus bovienii* XbSw_CS03	*Pseudomonas protegens* PpSw_SW4	*Pseudomonas protegens* PpSw_TCH07 2-2	*Pseudomonas putida* PpuSw_SW5	*Stenotrophomonas maltophilia* StmSw_SW1	*Ochrobactrum anthropi* OchaSw_SW2
Xenorhabdus nematophila XnSc_F1	++	**ND**	**-**	**-**	**p**	**+**	**-**	**-**	-	-	+	ND	-	-	-	-	++	-	+
Xenorhabdus poinarii XpSg_G6	++	++	-	-	-	-	-	-	**ND**	**-**	**-**	**-**	**-**	-	-	-	-	-	+
Xenorhabdus bovienii XbSw_CS03	+	+	-	-	-	-	-	-	-	-	-	ND	-	**ND**	**-**	**-**	**-**	**-**	**+**

Antibiosis was monitored by measuring the inhibition zones around colonies of producer strains overlaid with agar containing the indicator strains. Inhibition zones were scored as follows: (-), no inhibition; (+), inhibition zone from 10 to 20 mm; (++), inhibition zone from 20 to 40 mm; (v), variable inhibition; (ND), not determined; (p), partial inhibition. The data shown are representative of at least 3 experiments. As a positive control, we checked that *Xenorhabdus* strains displayed antimicrobial activity against the usual indicator strain, *Micrococcus luteus* ML. For producer and indicator strains recovered from the same *Steinernema* species, the results are in framed bold.

B.

	Positive control	*S carpocapsae* SK27 microbiota							*S glaseri* SK39 microbiota					*S weiseri* 583 microbiota					
Indicator strains / Producer strains	*Micrococcus luteus* ML	*Xenorhabdus nematophila* XnSc_F1	*Pseudomonas protegens* PpSc_PP-SC-10	*Achromobacter sp* AchSc_D7-1	*Alcaligenes faecalis* AlcfSc_SC	*Ochrobactrum sp* OchSc_ALL4	*Pseudochrobactrum* PochSc_AL3	*Stenotrophomonas* StSc_ALL5	*Xenorhabdus poinarii* XpSg_G6	*Pseudomonas protegens* PpSg_SG6 Apo	*Pseudomonas chlororaphis* PcSg_SK39 ApoA	*Stenotrophomonas maltophilia* StmSg_SK39-2	*Achromobacter* sp AchSg_SK39 ApoC	*Xenorhabdus bovienii* XbSw_CS03	*Pseudomonas protegens* PpSw_SW4	*Pseudomonas protegens* PpSw_TCH07 2-2	*Pseudomonas putida* PpuSw_SW5	*Stenotrophomonas maltophilia* StmSw_SW1	*Ochrobactrum anthropi* OchaSw_SW2
Pseudomonas protegens PpSc_PP-SC-10	v	v	ND	+	+	++	+	-	-	+	-	+	+	-	+	-	-	+	++
Pseudomonas protegens PpSg_SG6 Apo	+	++	-	+	-	+	+	+	+	ND	-	+	-	-	-	-	++	+	++
Pseudomonas chlororaphis PcSg_SK39 ApoA	-	v	+	v	++	-	-	-	-	-	ND	-	-	-	+	-	-	+	-
Pseudomonas protegens PpSw_SW4	v	++	+	+	+	++	+	++	-	-	-	+	+	+	ND	-	-	+	++
Pseudomonas protegens PpSw_TCH07 2-2	++	v	+	+	++	++	+	+	-	-	-	++	+	++	+	ND	-	+	++
Pseudomonas protegens CHAO^T	v	v	+	v	++	++	+	+	+	-	+	-	-	-	+	-	-	ND	+
Pseudomonas chlororaphis CFBP 2132^T	-	v	+	v	+	++	+	+	-	-	+	-	-	-	+	-	-	+	-

Antibiosis was monitored by measuring the inhibition zones around colonies of producer strains overlaid with agar containing the indicator strains. Inhibition zones were scored as follows: (-), no inhibition; (+), inhibition zone from 10 to 20 mm; (++), inhibition zone from 20 to 40 mm; (v), variable inhibition; (ND), not determined. The data shown data are representative of at least 3 experiments. As a positive control, we checked that *Pseudomonas* strains displayed antimicrobial activity against the usual indicator strain *Micrococcus luteus* ML. For producer and indicator strain recovered from the same *Steinernema* species, , the results are framed in bold.
