# Supplementary material for: Entomopathogenic nematode-associated microbiota: from monoxenic paradigm to pathobiome
Source: Microbiome. 2020 Feb 24;8:25. doi: 10.1186/s40168-020-00800-5 (PMC7041241; doi:10.1186/s40168-020-00800-5)
Supplement: Supplementary file 11 — Additional file 11. Bacterial strains isolated in the study and used in pathology and/or antibiosis assays. [file 40168_2020_800_MOESM11_ESM.pdf]

Additional File 11. Bacterial strains isolated in the study and used in pathology and/or antibiosis assays.

Species	Strain	Use	Source of isolation*	Nematode species if appropriate	Source	References
Pseudomonas protegens	CHAO^{T}	Pathology, antibiosis	Tobacco	-	A. Jousset	[1,2]
	PpSg_SG6 Apo	Pathology, antibiosis	Nematode (I)	*S. glaseri* SK39	DGIMI collection	this study
	PpSw_SW4	Pathology, antibiosis	Nematode (I)	*S. weiseri* 583	DGIMI collection	this study
	PpSw_TCH07 2-2	Pathology, antibiosis	Nematode (II)	*S. weiseri* 583	DGIMI collection	this study
	PpSc_PP-SC-10	Pathology, antibiosis	Nematode (III)	*S. carpocapsae* SK27	DGIMI collection	this study
Pseudomonas chlororaphis	PcSg_SK39 ApoA	Pathology, antibiosis	Nematode (I)	*S. glaseri* SK39	DGIMI collection	this study
	CFBP 2132^{T}	Pathology, antibiosis	Plate contaminant	-	O. Berge	[3,4]
Pseudomonas putida	PpuSw_SW5	Antibiosis	Nematode (I)	*S. weiseri* 583	DGIMI collection	this study
Stenotrophomonas maltophilia	StmSg_SK39-2	Pathology, antibiosis	Nematode (I)	*S. glaseri* SK39	DGIMI collection	this study
	StmSw_SW1	Pathology, antibiosis	Nematode (I)	*S. weiseri* 583	DGIMI collection	this study
	StmSw_TCH07 2-3	Pathology	Nematode (II)	*S. weiseri* 583	DGIMI collection	this study
	StmSc_ALL5	Pathology, antibiosis	Nematode (III)	*S. carpocapsae* All	DGIMI collection	this study
Achromobacter sp.	AchSc_D7-1	Antibiosis	Nematode (III)	*S. carpocapsae* SK27	DGIMI collection	this study
	AchSg_SK39 ApoC	Antibiosis	Nematode (I)	*S. glaseri* SK39	DGIMI collection	this study
Alcaligenes faecalis	AlcfSc_SC	Pathology, antibiosis	Nematode (III)	*S. carpocapsae* SK27	DGIMI collection	this study
Ochrobactrum sp.	OchSc_ALL4	Pathology, antibiosis	Nematode (III)	*S. carpocapsae* All	DGIMI collection	this study
Ochrobactrum anthropi	OchaSw_SW2	Pathology, antibiosis	Nematode (I)	*S. weiseri* 583	DGIMI collection	this study
	OchaHb_B3	Pathology	Nematode (III)	*H. bacteriophora* TT01	DGIMI collection	this study
Pseudochrobactrum sp.	PochSc_AL3	Pathology, antibiosis	Nematode (III)	*S. carpocapsae* All	DGIMI collection	this study
Xenorhabdus nematophila	XnSc_F1	Pathology, antibiosis	Nematode (II)	*S. carpocapsae* SK27	DGIMI collection	[5]
Xenorhabdus poinarii	XpSg_G6^{T} (ATCC49121)	Pathology, antibiosis	Nematode (II)	*S. glaseri* SK39	DGIMI collection	[6,7]
Xenorhabdus bovienii	XbSw_CS03	Pathology, antibiosis	Nematode (II)	*S. weiseri* 583	DGIMI collection	[8]
Photorhabdus luminescens	PholHb_TT01	Pathology	Nematode (III)	*H. bacteriophora* TT01	DGIMI collection	[9]
Escherichia coli	CIP 7624	Pathology	Clinical isolate	-	Institut Pasteur Collection	[10]
Micrococcus luteus	CIP 103430	Antibiosis	-	-	DGIMI collection	[11]

* mode of isolation is indicated between parentheses as follows:
I: isolated from *Galleria mellonella* after IJ infestation
II: isolated from IJ by the hanging drop technique [12]
III: isolated after IJ crushing

1. Flury P, Aellen N, Ruffner B, Péchy-Tarr M, Fataar S, Metla Z, et al. Insect pathogenicity in plant-beneficial pseudomonads: phylogenetic distribution and comparative genomics. ISME J. 2016.
2. Jousset A, Schuldes J, Keel C, Maurhofer M, Daniel R, Scheu S, et al. Full-Genome Sequence of the Plant Growth-Promoting Bacterium Pseudomonas protegens CHA0. Genome Announc. 2014;2.

3. Delorme S, Lemanceau P, Christen R, Corberand T, Meyer J-M, Gardan L. *Pseudomonas lini* sp. nov., a novel species from bulk and rhizospheric soils. Int J Syst Evol Microbiol. 2002;52:513–23.
4. Achouak W, Sutra L, Heulin T, Meyer JM, Fromin N, Degraeve S, et al. *Pseudomonas brassicacearum* sp. nov. and *Pseudomonas thivervalensis* sp. nov., two root-associated bacteria isolated from *Brassica napus* and *Arabidopsis thaliana.* Int J Syst Evol Microbiol. 2000;50:9–18.
5. Akhurst RJ, Boemare NE. A numerical taxonomic study of the genus Xenorhabdus (Enterobacteriaceae) and proposed elevation of the subspecies of X. nematophilus to species. J Gen Microbiol. 1988;134:1835–45.
6. Akhurst RJ. *Xenorhabdus nematophilus* subsp. *poinarii*: Its Interaction with Insect Pathogenic Nematodes. System Appl Microbiol. 1986;8:142–7.
7. Ogier J-C, Pages S, Bisch G, Chiapello H, Médigue C, Rouy Z, et al. Attenuated Virulence and Genomic Reductive Evolution in the Entomopathogenic Bacterial Symbiont Species, *Xenorhabdus poinarii*. Genome Biol Evol. 2014;6:1495–513.
8. Bisch G, Pages S, McMullen JG, Stock SP, Duvic B, Givaudan A, et al. *Xenorhabdus bovienii* CS03, the bacterial symbiont of the entomopathogenic nematode *Steinernema weiseri*, is a non-virulent strain against lepidopteran insects. J Invertebr Pathol. 2015;124:15–22.
9. Duchaud E, Rusniok C, Frangeul L, Buchrieser C, Givaudan A, Taourit S, et al. The genome sequence of the entomopathogenic bacterium *Photorhabdus luminescens*. Nat Biotechnol. 2003;21:1307–13.
10. Coyle MB, Lampe MF, Aitken CL, Feigl P, Sherris JC. Reproducibility of control strains for antibiotic susceptibility testing. Antimicrob Agents Chemother. American Society for Microbiology (ASM); 1976;10:436–40.
11. Boemare NE, Thaler JO, Lanois A. Simple bacteriological tests for phenotypic characterization of *Xenorhabdus* and *Photorhabdus* phase variants. Symbiosis. 1997;22:167–75.
12. Poinar GO. The Presence of *Achromobacter Nematophilus* in the Infective Stage of a *Neoaplectana* Sp. (Steinernematidae: Nematoda). Nematologica. 1966;12:105–8.
