# Supplementary material for: Entomopathogenic nematode-associated microbiota: from monoxenic paradigm to pathobiome
Source: Microbiome. 2020 Feb 24;8:25. doi: 10.1186/s40168-020-00800-5 (PMC7041241; doi:10.1186/s40168-020-00800-5)
Supplement: Supplementary file 12 — Additional file 12. List of the 62 bacterial strains, including 50 bacterial isolates from S.carpocapsae IJs, used in this study to build the 16S rRNA gene phylogenetic tree. [file 40168_2020_800_MOESM12_ESM.pdf]

Additional File 12: List of 62 bacterial strains including 50 bacterial isolates from *S.carpocapsae* IJs, used in this study to build the 16S rRNA gene phylogenetic tree

Strains	Source of isolation *	Species	*Steinernema carpocapsae* IJs	Multiplication batch (dd_mm_yy)	16S rRNA gene accession number
ATCC9220		*Achromobacter xylosoxidans*	-		AF411021
AchSc_D	Nematode (III)	*Achromobacter sp.*	SK27	20_07_16	MH810316
AchSc_D7-1	Nematode (III)				MH810317
AchSc_DD44	Nematode (III)		DD136_DGIMI	24_04_16	MH810319
AchSc_O-Bis	Nematode (III)		EGY03 (Egypt2)	20_07_16	MH810318
NCIB8687		*Alcaligenes faecalis*	-		AX110839
AlcSc_C3	Nematode (III)	*Alcaligenes sp.*	SK27	03_08_16	MH810329
AlcSc_ZZ	Nematode (III)			15_03_16	MH810332
AlcSc_SC	Nematode (III)			20_07_16	MK095583
AlcSc_D2	Nematode (III)				MH815099
AlcSc_AF	Nematode (III)				MH810337
AlcSc_AL1	Nematode (III)		All_DGIMI	01_06_16	MH810336
DSM7226^T		*Brevundimonas vesicularis*	-		LN681560
BreSc_P-Bis	Nematode (III)	*Brevundimonas sp.*	EGY03 (Egypt2)	20_07_16	MH810321
BreSc_ALL3	Nematode (III)		All_USDA	07_11_16	MH810323
BreSc_AL2	Nematode (III)		All_DGIMI	01_06_16	MH810322
BreSc_DD2	Nematode (III)		DD136_USDA	07_11_16	MH810325
LMG3301^T		*Ochrobactrum intermedium*	-		U70978
OchSc_ALL4	Nematode (III)	*Ochrobactrum sp.*	All_USDA	07_11_16	MK095934
PochSc_AL3	Nematode (III)	*Pseudochrobactrum sp.*	All_DGIMI	01_06_16	MH813437
CFBP2466^T		*Pseudomonas aeruginosa*	-		AF094713
CFBP2437^T		*Pseudomonas alcaligenes*	-		Z76653
CFBP2102^T		*Pseudomonas fluorescens*	-		AF094725
CFBP2066^T		*Pseudomonas putida*	-		AF094736
CFBP1392^T		*Pseudomonas syringae*	-		D84026
CHAO^T	Tobacco	*Pseudomonas protegens*	-		AJ278812
PpSc_PP-SC-10	Nematode (III)		SK27	10_05_16	MK095608
PpSc_C2	Nematode (III)			03_08_16	MH813482
PpSc_C5	Nematode (III)				MH813481
PpSc_SK27-col2	Nematode (III)		SK27_Toulouse	17_07_17	MH813534
PpSc_APO4	Nematode (III)		SK27_ApoII	07_11_16	MH813483
PpSc_APO6	Nematode (III)				MH813484
PpSc_APO7	Nematode (III)				MH813535
PpSc_B10-1	Nematode (III)		B10 (FRA36)	27_04_16	MH813533
PSc_D3	Nematode (III)	*Pseudomonas sp.*	SK27	20_07_16	MH814600
PSc_D5	Nematode (III)				MH814599
PSc_C	Nematode (III)				MH814601
PSc_C1	Nematode (III)			03_08_16	MH814637
PSc_C4	Nematode (III)				MH814598
PSc_D3-Apo	Nematode (I)		SK27_ApoII	01_04_14	MH814639
PSc_E2-Apo	Nematode (I)				MH814720
PSc_ALL1	Nematode (III)		All_USDA	07_11_16	MH814635
PSc_DD1	Nematode (III)		DD136_USDA	07_11_16	MH814645
PSc_DD3	Nematode (III)				MH814643
PSc_DD10	Nematode (III)		DD136_DGIMI	24_04_16	MH815093
PSc_DD22	Nematode (III)				MH814644
PSc_DD33	Nematode (III)				MH814721
ATCC13637^T		*Stenotrophomonas maltophilia*	-		AB008509
StmSc_D1	Nematode (III)		SK27	20_07_16	MH814356
StmSc_SK27-col6	Nematode (III)		SK27_Toulouse	17_07_17	MH813980
StmSc_APO2	Nematode (III)		SK27_ApoII	07_11_16	MH813997
StmSc_B2	Nematode (III)			01_04_14	MH813996
StmSc_C1-Apo2	Nematode (I)				MH814358
StmSc_B163	Nematode (I)			year 2014	MH813956
StmSc_ALL5	Nematode (III)		All_USDA	07_11_16	MH813995

ATCC19061[T]	Nematode (II)	*Xenorhabdus nematophila*	-		D78009
XnSc_F1	Nematode (II)		SK27	anterior to 1999	MH800287
XnSc_USGA01	Nematode (II)		All_DGIMI	14_05_09	MH807545
XnSc_USGA03	Nematode (II)		DD136_DGIMI	14_05_09	MH807543
XnSc_FR36	Nematode (II)		B10 (FRA36)	20_03_07	MH807542
XnSc_EG03	Nematode (III)		EGY03 (Egypt2)	20_07_16	MH807541
XnSc_FR241	Nematode (I)		GRAB (FRA241)	22_12_17	MH807544

* mode of isolation is indicated between parentheses, as follows:
I: isolated from *Galleria mellonella* after IJ infestation
II: isolated from IJ by the hanging drop technique [1]
III: isolated after IJ crushing

1. Poinar GO. The Presence of *Achromobacter Nematophilus* in the Infective Stage of a *Neoaplectana* Sp. (Steinernematidae: Nematoda). Nematologica. 1966;12:105–8.
